# Multi-Nutrient Analysis of Dietary Macronutrients with All-Cause, Cardiovascular, and Cancer Mortality: Data from NHANES 1999–2014

**DOI:** 10.3390/nu15020345

**Published:** 2023-01-10

**Authors:** Nicholas A. Koemel, Alistair M. Senior, David S. Celermajer, Amanda Grech, Tim P. Gill, Stephen J. Simpson, David Raubenheimer, Michael R. Skilton

**Affiliations:** 1 Charles Perkins Centre, The University of Sydney, Sydney 2006, Australia; 2 Sydney Medical School, The University of Sydney, Sydney 2006, Australia; 3 School of Life and Environmental Sciences, The University of Sydney, Sydney 2006, Australia; 4 Susan Wakil School of Nursing and Midwifery, The University of Sydney, Sydney 2006, Australia

**Keywords:** diet, macronutrients, mortality, cardiovascular disease, cancer, NHANES

## Abstract

Macronutrients are a major component of the human diet. However, few studies have assessed their collective association with mortality. We sought to evaluate the associations of macronutrient intake with all-cause, cardiovascular, and cancer mortality in US adults using a multi-nutrient approach. This prospective cohort analysis used data from the National Health and Nutrition Examination Survey from the years 1999 to 2014. The participants included 33,681 US adults aged 20–85 years (52.5% female). The maximum follow-up time was 16.8 years, with a total of 4398 total deaths, including 772 cardiovascular deaths and 952 cancer deaths. The associations between mortality and dietary macronutrients were explored using three-dimensional generalized additive models, allowing for visual and statistical inference of complex nonlinear associations. Absolute macronutrient intake demonstrated a three-way interactive association with all-cause mortality (*p* < 0.001), cardiovascular mortality (*p* = 0.02), and cancer mortality (*p* = 0.05), adjusted for age, sex, ethnicity, socioeconomic status, dietary quality, and lifestyle. Compositionally, a high caloric diet composed of moderately high protein (20%), moderate fat (30%), and moderate carbohydrate (50%) levels was associated with the highest mortality risk. Across the total energy intake levels, lower mortality risk was observed in two separate regions consisting of higher protein (30%), higher carbohydrate (60%), and lower fat levels (10%) or lower protein (10%), moderate carbohydrate (45%), and higher fat levels (45%). These findings highlight a complex nonlinear and interactive association between macronutrients and all-cause mortality such that several distinct dietary compositions are associated with similarly high or low risk. Future research is needed to explore the drivers of these associations and whether they differ across varying dietary patterns and populations.

## 1. Introduction

Human nutrition is a powerful risk factor for the development of numerous non-communicable diseases, including cardiovascular disease and some cancers [1,2,3]. Collectively, non-communicable diseases remain the leading cause of death in the United States [4]. A large body of nutritional research has focused on individual nutrient components such as macronutrients, fatty acids, fiber, and added sugar, although assessment of individual nutrients is unlikely to fully capture the impact of diet on disease risk and mortality [5,6]. A recent analysis of the National Health and Nutrition Examination Survey (NHANES) from the years 1999 to 2014 found no association between diet based on macronutrient intakes, specifically low carbohydrate and low-fat diets, and all-cause or cause-specific mortality [7]. Alternatively, the UK Biobank revealed that many of the associations for macronutrient intake demonstrate a nonlinear relationship with all-cause mortality [8]. For example, carbohydrate intake showed no association when comprising 20–50% of the diet but a positive association with all-cause mortality at 50–70% of intake. These findings align with a recent multinational meta-analysis of seven cohorts, which revealed a nonlinear U-shaped association for absolute carbohydrate intake with all-cause mortality [9]. An underlying complexity for the relationship between macronutrients and mortality appears to exist, supporting the need to evaluate the intake of dietary macronutrients collectively.

Nutritional geometry is a tool that has been applied in both animal models [10,11] and human studies [12,13,14] to better understand the relationships between diet composition, physiology, and health. In contrast to methods which examine the associations of individual nutrients with outcomes, this approach allows for outcomes to be mapped across a multi-dimensional nutrient space, thus enabling the analysis and visualizations of nonlinear associations and interactions. Recently, this approach has been applied to country-level population data to describe the associations of macronutrients with age-specific mortality [15]. However, this technique has yet to be applied to individual participant-level data to describe the associations of macronutrients with all-cause mortality.

Accordingly, we sought to assess the relationship between macronutrient intake with all-cause mortality using a nutritional geometry approach. As secondary outcomes, we explored the relationship between macronutrient intake and cardiovascular and cancer mortality. We hypothesized that there would be a complex association of macronutrients with all-cause mortality characterized by nonlinear and interactive elements that would not be detected in the analysis of individual nutrients. Identifying such relationships is important for directing future research into the links between diet and mortality and informing interventions for improved health.

## 2. Materials and Methods

### 2.1. Study Population

This analysis was undertaken using the NHANES’s annually collected cross-sectional survey completed by the National Center for Health Statistics to assess dietary intake and health-related outcomes in the United States. The NHANES study protocol was approved by the ethics review board of the National Center for Health Statistics, and all data were made publicly available. We reviewed the NHANES data from 1999 to 2014 for individuals older than 20 years of age (Figure S1). Dietary data were collected at the initial interview via two separate 24 h dietary recalls recorded by a trained nutrition professional and assessed as the average of both recalls. The first recall was collected in person at a mobile examination center, and the second interview was conducted by telephone 3–10 days later. During the years 1999–2002, only a single 24 h dietary recall was collected at the initial examination [16]. To correct for measurement error, individuals with a minimum of two 24 h recalls were adjusted using a validated multiple system method to reflect the habitual intake of nutrients [17,18].

Mortality data were ascertained via data linkage to the National Death Index. Cause-specific mortality was defined using the underlying cause of death codes per the International Statistical Classification of Diseases and Related Health Problems, Tenth Revision (ICD-10) [19]. Cardiovascular mortality was classified as all deaths related to the circulatory system (I00–I09, I11, I13, and I20–I51), while cancer mortality included all cancer types (C00–C97). Survival data were assessed as the follow-up time in years from the examination, and in cases where censor data were missing, the participants were assumed to be alive.

### 2.2. Demographic and Lifestyle Covariates

Demographic and lifestyle covariates including sex (male or female) were self-reported. Participants who reported their race or ethnicity were categorized as either non-Hispanic white, non-Hispanic black, Hispanic, or other. Education was categorized as less than high school, high school, or some college and above. Household income was included as the ratio of family income to poverty. Smokers were defined as individuals who reported smoking 100 cigarettes in their lifetime. Alcohol consumers were classified as those who drank a minimum of 12 drinks within any given year. Physical activity was calculated as the self-reported metabolic equivalents of moderate-to-vigorous leisure activity completed weekly. The body mass index (BMI) was measured as weight in kilograms divided by height in meters squared. Dietary quality was evaluated using the Healthy Eating Index, which assesses adherence to the 2015–2020 US Dietary Guidelines with a score ranging from 0–100 possible points [20].

### 2.3. Statistical Analyses

For the primary analysis, we excluded individuals with potential over- or under-reporting for dietary energy intake, which included males <800 or >4200 kcal/day and females <600 or >3500 kcal/day (*n* = 9671). Individuals with macronutrient intakes 3 standard deviations from the mean were not included in the analysis (*n* = 442).

Associations between dietary macronutrients and mortality were analyzed using generalized additive models (GAMs). GAMs are a form of multivariable regression that allows for visual and statistical assessment of nonlinear associations. Macronutrient exposures were assessed using three-dimensional smooth terms of macronutrients. Such smooth terms can default to simplistic linear regression coefficients (e.g., in a generalized linear model), where such a relationship is deemed to be the best fit for the data, but also allow for more complex nonlinear relationships. GAMs are flexible in that they can account for different data types via link functions and the specification of model families. Here, we used GAMs to implement a Cox proportional hazard model for mortality. As with conventional multiple regression, which is widely used in epidemiology, GAMs can make statistical corrections for confounding variables through the inclusion of covariates. Model one was the base model and included adjustments for age, sex, and household income (Table S1). Model two was adjusted as per model one with further adjustment for sociodemographic characteristics, specifically race or ethnicity and education (Table S2). Model three was adjusted as per model two with further adjustments for lifestyle habits, specifically alcohol intake, smoking, BMI, physical activity, and the Healthy Eating Index (Table S3).

GAMs were implemented using the “gam” function from the *mgcv* package in R statistical software and estimated using generalized cross-validation (v. 1.8-31; R Core Team; Vienna, Austria) [21,22]. The three-dimensional effects of nutrition as estimated by the GAMs were visualized as response surfaces. Three response surfaces were plotted for each outcome, with each surface plotted on two macronutrient exposures expressed for the absolute intake (kcal/day) as the *x*- and *y*-axes, while the third macronutrient was held constant at the 25th, 50th, and 75th percentile of its intake. Colored surfaces spanning this nutrient space represent mortality risk as a survival function for predicting the mean survival follow-up time of those included in the analysis. A significant macronutrient model indicates that the association of each macronutrient with the specified outcome is dependent upon the relative intake of the other two macronutrients. Given the complex nonlinearity in these models, specific macronutrient relationships and effect sizes must be interpreted visually. The current *mgcv* package does not allow for the inclusion of survey weights that account for the complex design of the NHANES, and thus findings cannot be generalized to the US population.

As well as presenting the results of GAMs on the absolute intake scale (kcal/day), we also transformed the GAM outputs to allow for inference about the macronutrient composition as a proportion of the total energy. Following transformation from the absolute intake to a proportion of energy, the GAM outputs were used to map mortality risk on the survival function scale onto a mixture triangle of macronutrient composition using the *ggplot2* package in R statistical software (v. 3.3.5; R Core Team; Vienna, Austria) [23]. The survival function scores can be interpreted as the probability that an individual survived longer than the mean survival time. We used right-angle mixture triangles, which enabled compositional inference by showing the percentage of dietary protein and carbohydrates on the *x*- and *y*-axes, while fat intake can be inferred for any point within the triangle by subtracting the sum of the proteins and carbohydrates from 100 [24]. These response surfaces of macronutrient composition and mortality were generated for different total energy intakes: the 25th, 50th, and 75th percentile of caloric intake. Response surfaces in the absolute and compositional models were adjusted for age, sex, income, Healthy Eating Index, BMI, and physical activity. The Cox proportional hazard assumptions were checked.

Mixture triangles were also used to visualize the association of dietary macronutrients with the Healthy Eating Index score and putative dietary confounders, such as the fatty acid profile and fiber, sugar, and sodium levels. The fatty acid profile was examined by plotting dietary polyunsaturated, saturated, or monounsaturated fat as a percentage of fat energy on a macronutrient mixture triangle. Fiber, sugar, and sodium levels were plotted as the average intake reported from the 24 h recalls.

### 2.4. Subgroup and Sensitivity Analyses

Subgroup analyses were undertaken to assess the associations in males and females using sex-specific GAMs and response surface plots. Potential interaction between macronutrients and sex was explored in the GAM models, with model comparisons made with the Akaike Information Criterion (AIC) [25] to compare the model fit with and without sex-stratified smoothing (using the “by” term in the “gam” function in *mgcv*). A difference in the AIC of >2 was considered a significant improvement in model fit and interpreted as evidence of a sex-specific effect of macronutrient intake on the mortality outcome. Several sensitivity analyses were conducted to explore potential limitations in this study. In females, we conducted a sensitivity analysis removing those pregnant during the examination or interview (*n* = 1008). To test for potential reverse causation from chronic diseases, a comorbidity sensitivity analysis excluded individuals who reported having hypertension, cardiovascular disease, or cancer or were recommended by a doctor to take anti-hypertensive, lipid-lowering, or glucose-controlling medication (*n* = 12,303). A sensitivity analysis was completed including only individuals who had two completed 24 h recalls. A follow-up sensitivity model was assessed by removing all individuals who died in the first year of follow-ups (*n* = 308).

## 3. Results

### 3.1. Participant Characteristics

The participant’s characteristics at enrollment are shown in Table 1. After excluding individuals with potential over- or under-reporting of dietary energy intake, this analysis included 33,681 US adults (49.7 ± 18.3 years; 52.5% female). The maximum follow-up time was 16.8 years (8.6 ± 5.3 years) with 4398 total deaths, 772 cardiovascular deaths, and 952 cancer deaths. Individuals who reported having comorbidities at the time of the interview included diabetes (*n* = 3826), cardiovascular disease (*n* = 3384), and cancer (*n* = 3068). Medication for related comorbid conditions included that for diabetes (*n* = 2831), hypertension (*n* = 7107), and lipid-lowering medications (*n* = 4325).

### 3.2. Macronutrients and All-Cause Mortality

**Figure 1:**
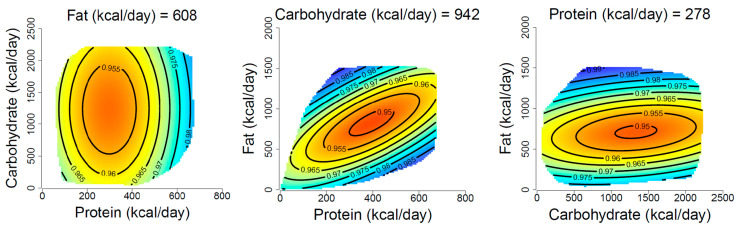
Macronutrient absolute intake and all-cause mortality. Each surface shows the survival function scale for all-cause mortality in a nutrient space of all three macronutrients. The *x* and *y*-axes represent two macronutrient exposures sliced through the median value of the macronutrient shown at the top of each figure. Response values are colored such that warm colors show a higher risk of mortality and cooler colors show a lower mortality risk. Response surfaces were adjusted for age, sex, household income, BMI, physical activity, and Healthy Eating Index.

Macronutrient intake demonstrated a three-way interactive association with all-cause mortality in the fully adjusted model (*p* < 0.001; Table 2). The associations of absolute macronutrient intake with all-cause mortality are shown in Figure 1 (extended figure in Figure S2; unadjusted response surface in Figure S3). The coefficients for models 1–3 are shown in Tables S1–S3. The response surfaces highlight a region of high mortality risk (i.e., low survivorship), shown in red near the center. This pattern indicates lower survival scores in those with a moderate intake of all-three macronutrients, with the highest survival shown in the outer regions of the response surfaces depending on the relative intake of protein and fat.

**Figure 2:**
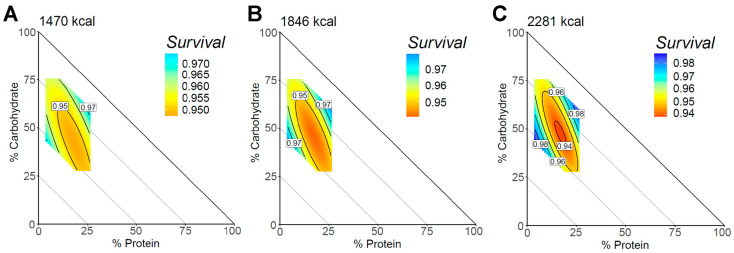
Macronutrient composition with all-cause mortality. The mixture triangle shows the model predictions of the all-cause mortality survival score for the range of macronutrient percentages in this dataset. (**A**–**C**) The predictions using the 25th, 50th, and 75th percentiles of caloric intake for the study population. The *x* and *y*-axes show protein and carbohydrate levels, respectively. Percentage of fat can be inferred as decreasing and moving away from the origin such that each point on the triangle can be summed to equal 100%. Response values are colored such that warm colors show a higher risk of mortality and cooler colors show a lower mortality risk. Response surfaces were adjusted for age, sex, household income, BMI, physical activity, and Healthy Eating Index.

When analyzed separately, carbohydrates and fat demonstrated nonlinear relationships with all-cause mortality (Figure S4; Table S3). The macronutrient composition and all-cause mortality are shown in Figure 2, and the unadjusted models are presented in Figure S5. The lowest survival scores appeared in a high caloric diet composed of moderately high protein (20%), moderate carbohydrate (50%), and moderate fat levels (30%). The highest survival scores were identified in a region of higher protein (30%), higher carbohydrate (60%), and lower fat levels (10%) and the region of lower protein (10%), moderate carbohydrate (45%), and higher fat levels (45%). This association was similar across energy intakes, with a higher overall effect size at higher energy intakes.

**Figure 3:**
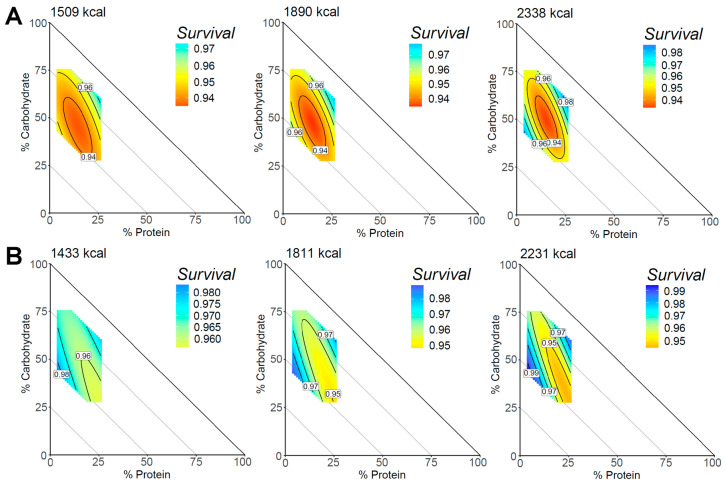
Associations of macronutrient composition with all-cause mortality for males and females. The mixture triangle shows the model predictions of the all-cause mortality survival score for the range of macronutrient percentages in this dataset. (**A**) The predictions using the 25th, 50th, and 75th percentiles of caloric intake for males, and (**B**) shows this for females. The *x* and *y*-axes show protein and carbohydrate levels, respectively. Percentage of fat can be inferred as decreasing and moving away from the origin such that each point on the triangle can be summed to equal 100%. Response values are colored such that warm colors show a higher risk of mortality and cooler colors show a lower mortality risk. Response surfaces were adjusted for age, sex, household income, BMI, physical activity, and Healthy Eating Index.

The sex-stratified associations with macronutrient composition and all-cause mortality are shown in Figure 3. When stratified by sex, and after full adjustment, the association of macronutrient intake with mortality was significant in males and females (Table 2). The response surfaces demonstrated sex-specific associations, where males showed lower overall survival scores. At lower energy intakes, males with diets composed of higher protein levels coupled with lower fat levels appeared to have higher survivability rates. Conversely, at lower energy intakes, females showed higher survival scores with diets composed of less protein coupled with more fat. Both sexes appeared to have similar high- and low-risk regions at higher energy intakes.

### 3.3. Macronutrients and Cardiovascular and Cancer Mortality

In the analyses of individual macronutrients in isolation, there was no association of carbohydrates, protein, or fat with cardiovascular and cancer mortality (Figure S4; Table S4). The relationship between collective macronutrient intake and cardiovascular and cancer mortality is shown in Figures S6 and S7, and the response surfaces shown at the 25th, 50th, and 75th percentiles of energy intake are shown in Figures S8 and S9. There was a significant three-way interaction for macronutrients with both cardiovascular mortality (*p* = 0.02) and cancer mortality in the fully adjusted models (*p* = 0.05; Table 2). The cardiovascular and cancer mortality survival scores were lowest with moderate protein consumption coupled with either moderate fat intake or high carbohydrate intake. The compositional mixture triangles support these findings such that lower survival scores appeared extending across a band of moderate fat intake (30%) with higher survivability at either higher protein or lower protein levels, depending on the relative intake of fat (Figures S10 and S11).

When stratified by sex, and after full adjustment, the association of macronutrient intake with cardiovascular mortality was statistically significant in males but not in females (Table 2; Figure S12). For males, the lowest survival scores were associated with lower-energy diets composed of less protein (5%), more carbohydrates (75%), and less fat (15%). There was no significant association for macronutrient intake with cancer mortality in the sex-stratified analysis (Table 2; Figure S13).

### 3.4. Other Sensitivity Analyses and Nutritional Correlates

There was no improvement in model fitness of the results when including sex as an interactive term for all-cause mortality. Cardiovascular and cancer mortality models without sex as an interactive term were favored by the AIC (Tables S5 and S6). The results did not differ for females when those who were pregnant at the time of dietary assessment were excluded (Table 2). When excluding those with comorbid conditions or taking related medications, the association of macronutrient composition with cancer mortality was no longer significant in the fully adjusted model (Figure S14; Table S7). Omitting individuals who did not complete two separate dietary recalls did not change the overall findings for all-cause mortality, but it did result in non-significant associations of macronutrients with cardiovascular and cancer mortality (Figure S15; Table S8). There were no major differences in the follow-up sensitivity except for cancer mortality, which was no longer significant in the fully adjusted model (Table 2; *p* = 0.09).

Response surface plots for the Healthy Eating Index scores, fatty acid subtypes, and fiber, sugar, and sodium levels are shown in Figures S16–S18. The Healthy Eating Index was higher in diets composed of more protein coupled with less fat. The fatty acid profile varied across the macronutrient composition and different total energy intakes. The percentage of fat derived from saturated fat was generally the highest while that for polyunsaturated fat was the lowest in diets composed of less protein, less fat, and more carbohydrate intake. The percentage of fat derived from monounsaturated fat was highest in diets composed of more carbohydrates coupled with moderate protein intake. Dietary fiber intake was the highest in those consuming a higher-protein, lower-carbohydrate, and higher-fat diet. Sugar intake was the highest in those consuming a high proportion of energy from carbohydrates and a low proportion from protein. The sodium level was the highest in those consuming a diet composed of more protein coupled with lower fat at a higher total energy intake.

## 4. Discussion

In this study, we applied a method known as nutritional geometry to analyze and visualize the collective association of macronutrients with all-cause, cardiovascular, and cancer mortality. Our findings demonstrate a complex association of macronutrients with all-cause mortality that is not captured by analysis of carbohydrate, fat, and protein levels separately. In the analyses in which macronutrients were expressed as a proportion of energy in the diet, we found evidence of both nonlinear associations and macronutrient–macronutrient interactions. We showed that diets higher in energy and composed of moderately high protein (20%), moderate carbohydrate (50%), and moderate fat levels (30%) were associated with the highest mortality risk. Compositional analyses of the intake of macronutrients revealed similar patterns across energy intakes, although the effect size was more pronounced at higher overall energy intakes.

This study provides additional insight concerning the relationships between macronutrients and all-cause mortality. Many epidemiological studies have examined the relationships between individual dietary macronutrients and metabolic health [26] and mortality [9,27,28]. When applying a single nutrient approach, our results were consistent with previous epidemiological findings. However, when we applied a methodology that could assess the associations of all three macronutrients simultaneously, we observed some surprising relationships between diet and predicted mortality.

For example, in our analysis of absolute nutrient intakes (Figure 1), we observed a higher risk of mortality to be associated with diets in which all macronutrients were consumed at intermediate levels, whereas previous studies suggested that high intake of protein, fat, and carbohydrates are all individually associated with higher mortality risk [9,29,30]. Likewise, our analysis of macronutrient composition showed that survival was lowest spanning across the full range of carbohydrate and fat intake coupled with a protein intake in the intermediate range of 15–25% of energy. Previous analysis in the same cohort (NHANES) revealed a U-shaped relationship with carbohydrate intake, where increased mortality risk was associated with diets composed of <40% carbohydrates [31]. Analysis of the UK Biobank revealed nonlinear associations of individual macronutrients with all-cause mortality [8]. Our findings when examining individual nutrients were consistent with these associations, with the lowest risk of all-cause mortality being at approximately 50% carbohydrate intake. When examined using a multi-nutrient approach, however, the results appeared to be more nuanced. For example, in the compositional analyses (Figure 2), a moderate intake of carbohydrates (approximately 50% energy) was associated not with the lowest risk but rather a range of risks of all-cause mortality, dependent on the intake of fat and protein. Similarly, low-carbohydrate and low-fat diets were both associated with a range of risks, dependent on the intake of the other macronutrients. McKenzie et al. investigated macronutrients compositionally using cluster analysis in the UK Biobank and found that diets composed of 19% protein, 45% carbohydrates, and 29% fat were associated with a lower risk of all-cause mortality [32]. Conversely, the present study found this region to be associated with a higher risk of mortality. These inconsistencies may be partially explained by participant demographics, with the UK Biobank having an older and healthier population than the NHANES [33].

There was no statistical evidence of an interaction between sex and macronutrients for all-cause, cardiovascular, or cancer mortality. Stratified analyses identified that the associations between macronutrients and all-cause mortality displayed unique sex-specific differences. The response surfaces for men and women identified different regions of macronutrient intake associated with the highest survival probability at lower energy intakes. For males with lower energy intake, a diet of more protein coupled with less fat had the highest survivability. Conversely, females with lower energy intake had the highest survivability with diets composed of less protein coupled with more fat. At higher energy intakes, both males and females had the highest survival scores, as seen in the pooled analysis, although the males had lower scores overall. This may be partly related to specific nutrient requirements and related diseases that differ for males and females throughout the course of life [34].

### Strengths and Limitations

A key strength of this study was that our approach enabled both statistical analysis and visual representation of the complex associations of all three macronutrients with mortality outcomes simultaneously. This multi-nutrient approach provides a novel perspective in the area of nutritional epidemiology by exploring the associations of diet composition collectively, rather than taking a one-variable-at-a-time approach.

However, the multi-nutrient approach used in this study provides a graphical representation of the associations of macronutrient composition with both cause-specific and all-cause mortality. This is met with the limitation that specific confidence intervals cannot be produced for each graphical point. Other limitations include those inherent to most dietary assessment methods applied in epidemiological research, such as the potential for misreporting dietary intake, in addition to dietary intake having only been assessed at a single time point during the course of life. Although data transformation is performed to reflect habitual intake, the 24 h recall often poorly describes an individual’s habitual diet. Additionally, both dietary habits and nutrient needs change dynamically through the course of life, which cannot be readily examined in a cross-sectional design. Taken together, these analyses were from an observational cohort, and as such, causality cannot be inferred.

The current US dietary guidelines do not define specific compositional recommendations for macronutrients, rather focusing on specific foods, food groups, and dietary patterns [35]. However, the Institute of Medicine (IOM) recommends maintaining an intake of 10–35% protein, 45–65% carbohydrates, and 20–35% fat [36], and the dietary guidelines of several other countries make similar recommendations [37,38,39,40,41]. These recommended ranges were proposed to minimize the incidence of non-communicable diseases and take into consideration the optimal macronutrient composition for reducing the intake of detrimental dietary components without compromising the ability to meet micronutrient requirements. In the present study, a macronutrient composition within the range recommended by the IOM included both areas of high and low risk of mortality within the surface plots.

In common with our study, many studies have focused on the relationships between dietary macronutrient quantity and health. However, other properties of diets, including macronutrient quality, dietary fiber, micronutrients, and non-nutritional components such as phytochemicals, also play a role [42,43,44,45]. Examining these in multi-dimensional models is an important priority, but that was beyond the scope of the present study. However, to test the robustness of our results, we adjusted the macronutrient models using the Healthy Eating Index, which is designed to be sensitive to overall diet quality by measuring compliance with the US dietary guidelines. Provided that this adjustment did not affect the conclusions, the findings suggest that the macronutrient associations we detected are robust and warrant more detailed exploration.

Lastly, it should be noted that this study was not sufficiently powered to explore specific types of cardiovascular mortality or specific cancer sites. Future studies should determine the relationship between dietary macronutrient composition and specific classifications of cardiovascular and cancer mortality.

## 5. Conclusions

In this study, macronutrient composition was significantly associated with all-cause, cardiovascular, and cancer mortality in a complex and unexpected manner involving nonlinear and interactive associations. Future research is needed to explore the mechanisms driving these relationships and how they differ across varying dietary patterns and populations.

## Figures and Tables

**Table 1:** Participant characteristics *.

Participant Characteristic	Mean	SD
Age (years)	49.7	18.4
Female Sex (%)	52.5	−
BMI (kg/m^2^)	28.8	6.5
Total Energy (kcal)	1907	603
Healthy Eating Index Score	52.7	12.8
Protein (kcal)	290	106
Protein (TEI%)	15.2	4.0
Carbohydrate (kcal)	978	340
Carbohydrate (TEI%)	51.4	7.3
Fat (kcal)	639	246
Fat (TEI%)	33.4	6.2
SFA (kcal)	219	93
SFA (FEI%)	36.0	5.2
PUFA (kcal)	149	63
PUFA (FEI%)	24.7	4.9
MUFA (kcal)	241	101
MUFA (FEI%)	39.3	3.3
Fiber (g)	16	7
Sugar (g)	78	8
Sodium (mg)	1469	81
Race or Ethnicity		
Hispanic (%)	25.7	−
Non-Hispanic White (%)	46.2	−
Non-Hispanic Black (%)	20.6	−
Other (%)	7.5	−
Family Income-to-Poverty Ratio	2.5	1.6
Education Level		
Less than High School (%)	10.2	−
High School Graduate or GED (%)	76.2	−
Some College or More (%)	13.6	−
Non-drinker (%)	26.5	−
Non-smoker (%)	50.4	−
Physical Activity (METs)	1657	2212

* Participant characteristics: Body Mass Index (BMI); percentage of total energy intake (TEI%); percentage of total fat intake (FEI%); and standard deviation (SD).

**Table 2:** Generalized additive models for macronutrient intake and mortality *.

Outcome	Model ^1^				Model ^2^				Model ^3^			
	Inc.	*n*	Dev Exp	*p*	Inc.	*n*	Dev Exp	*p*	Inc.	*n*	Dev Exp	*p*
**All-Cause Mortality**	4398	33,681	26.9%	<0.001	4398	33,681	27.1%	<0.001	4398	33,681	28.1%	<0.001
Males	2412	15,993	25.3%	<0.001	2412	15,993	25.5%	<0.001	2412	15,993	26.4%	<0.001
Females	1986	17,688	28.0%	<0.001	1986	17,688	28.1%	<0.001	1986	17,688	29.5%	<0.001
Pregnancy Sensitivity	1961	16,680	27.2%	<0.001	1961	16,680	27.3%	<0.001	1961	16,680	28.8%	<0.001
Comorbid Sensitivity	1455	21,378	26.3%	<0.001	1455	21,378	26.5%	<0.001	1455	21,378	27.1%	<0.001
Dietary Recall Sensitivity	1789	20,339	23.4%	0.04	1789	20,339	23.7%	0.03	1789	20,339	25.1%	0.03
Follow-up Sensitivity	4090	33,358	27.2%	<0.001	4090	33,358	27.4%	<0.001	4090	33,358	28.2%	<0.001
**Cardiovascular Mortality**	772	30,055	31.8%	0.03	772	30,055	32.0%	0.02	772	30,055	33.1%	0.02
Males	468	14,049	28.6%	0.03	468	14,049	28.8%	0.03	468	14,049	30.2%	0.04
Females	304	16,006	36.0%	0.12	304	16,006	36.1%	0.10	304	16,006	37.8%	0.09
Pregnancy Sensitivity	303	15,022	35.0%	0.15	303	15,022	35.2%	0.12	303	15,022	37.0%	0.11
Comorbid Sensitivity	207	20,130	34.7%	0.14	207	20,130	34.7%	0.12	207	20,130	36.1%	0.05
Dietary Recall Sensitivity	302	18,852	24.8%	0.93	302	18,852	25.0%	0.94	302	18,852	26.8%	0.93
Follow-up Sensitivity	699	29,967	32.1%	0.06	699	29,967	32.3%	0.04	699	29,967	33.3%	0.05
**Cancer Mortality**	952	30,235	21.0%	0.04	952	30,235	21.3%	0.03	952	30,235	22.3%	0.05
Males	557	14,138	25.2%	0.39	557	14,138	23.8%	0.29	557	14,138	24.5%	0.31
Females	395	16,097	16.7%	0.08	395	16,097	16.8%	0.07	395	16,097	17.5%	0.11
Pregnancy Sensitivity	388	15,107	15.9%	0.09	388	15,107	16.0%	0.08	388	15,107	16.7%	0.12
Comorbid Sensitivity	351	20,274	21.2%	0.13	351	20,274	21.4%	0.10	351	20,274	22.0%	0.11
Dietary Recall Sensitivity	391	18,941	16.4%	0.27	391	18,941	16.7%	0.24	391	18,941	17.8%	0.24
Follow-up Sensitivity	878	30,146	21.0%	0.09	878	30,146	21.2%	0.07	878	30,146	22.1%	0.09

* Models 1–3 sequentially adjust for various covariates. The table reflects the incidence of mortality (Inc.), deviance explained (Dev Exp) by the entire model, and the *p* value for macronutrients as a three-dimensional smooth term. A significant macronutrient model can be interpreted such that the relationship of each macronutrient with the specified outcome is dependent upon the relative intake of all three macronutrients. Given the complex nonlinearity in these models, specific macronutrient relationships and effect sizes are best interpreted visually.

## Data Availability

Data from the National Health and Nutrition Examination Survey are publicly available online at https://www.cdc.gov/nchs/nhanes/index.htm (accessed on 15 May 2021).

## Supplementary Materials

The following supporting information can be downloaded at: https://www.mdpi.com/article/10.3390/nu15020345/s1, Figure S1: Participant Flowchart; Figure S2: Associations of Absolute Macronutrient Intake with All-Cause Mortality at the 25th, 50th, and 75th Percentile of Intake for Each Macronutrient; Figure S3: Unadjusted Absolute Macronutrient Intake and All-Cause Mortality; Figure S4: Individual Macronutrient Associations with All-Cause, Cardiovascular, and Cancer Mortality; Figure S5: Unadjusted Macronutrient Composition and All-Cause Mortality; Figure S6: Absolute Macronutrient Intake and Cardiovascular Mortality; Figure S7: Absolute Macronutrient Intake and Cancer Mortality; Figure S8: Associations of Absolute Macronutrient Intake with Cardiovascular Mortality at the 25th, 50th, and 75th Percentile; Figure S9: Associations of Absolute Macronutrient Intake with Cancer Mortality at the 25th, 50th, and 75th Percentile of Intake for Each Macronutrient; Figure S10: Macronutrient Composition and Cardiovascular Mortality; Figure S11: Macronutrient Composition and Cancer Mortality; Figure S12: Macronutrient Composition and Cardiovascular Mortality for Males and Females; Figure S13: Macronutrient Composition and Cancer Mortality for Males and Females; Figure S14: Macronutrient Composition and All-Cause Mortality: Comorbidity Sensitivity Analysis; Figure S15: Macronutrient Composition and All-Cause Mortality: Sensitivity Analysis Including Only Participants with Two Complete 24-h recalls; Figure S16: Macronutrient Composition and Healthy Eating Index; Figure S17: Macronutrient Composition and Dietary Fatty Acid Profile; Figure S18: Macronutrient Composition and intake of Dietary Fiber, Sugar, and Sodium. Table S1: Model^1^ Generalized Additive Model Coefficients for Macronutrient Intake and Mortality; Table S2: Model^2^ Generalized Additive Model Coefficients for Macronutrient Intake and Mortality; Table S3: Model^3^ Generalized Additive Model Coefficients for Macronutrient Intake and Mortality; Table S4: Generalized Additive Model Coefficients for Individual Macronutrient Percentages and Mortality; Table S5: Generalized Additive Model Coefficients for Macronutrient Intake and Mortality with Interaction for Sex; Table S6: Model Comparisons for With and Without Macronutrients by Sex Interaction; Table S7: Generalized Additive Model Coefficients for Comorbidity Sensitivity; Table S8: Generalized Additive Model Coefficients for Dietary Recall Sensitivity.
