# Supplementary material for: Multi-Nutrient Analysis of Dietary Macronutrients with All-Cause, Cardiovascular, and Cancer Mortality: Data from NHANES 1999–2014
Source: Nutrients. 2023 Jan 10;15(2):345. doi: 10.3390/nu15020345 (PMC9865351; doi:10.3390/nu15020345)
Supplement: Supplementary file 1 [file nutrients-15-00345-s001.zip › nutrients-2119477-supplementary.pdf]

ONLINE SUPPLEMENTARY MATERIAL

Multi-nutrient analysis of dietary macronutrients with all-cause, cardiovascular, and cancer mortality: data from NHANES 1999-2014

Nicholas A. Koemel[1,2]; Alistair M. Senior[1,3]; David S. Celermajer[2]; Amanda Grech[1,3]; Tim P. Gill[1,2,4]; Stephen J. Simpson[1,3]; David Raubenheimer[1,3]; Michael R. Skilton[1,2]

[1]Charles Perkins Centre, The University of Sydney, Sydney 2006, Australia

[2]Sydney Medical School, The University of Sydney, Sydney 2006, Australia

[3]School of Life and Environmental Sciences, The University of Sydney, Sydney 2006, Australia

[4]Susan Wakil School of Nursing and Midwifery, The University of Sydney, Sydney 2006, Australia

TABLE OF CONTENTS

Page

I. SUPPLEMENTARY FIGURES

Participant Flowchart 3
Associations of Absolute Macronutrient Intake with All-Cause Mortality at the 25th, 50th, and 75th Percentile of Intake for Each Macronutrient 4
Unadjusted Absolute Macronutrient Intake and All-Cause Mortality 5
Individual Macronutrient Associations with All-Cause, Cardiovascular, and Cancer Mortality 6
Unadjusted Macronutrient Composition and All-Cause Mortality 7
Absolute Macronutrient Intake and Cardiovascular Mortality 8
Absolute Macronutrient Intake and Cancer Mortality 9
Associations of Absolute Macronutrient Intake with Cardiovascular Mortality at the 25th, 50th, and 75th Percentile of Intake for Each Macronutrient 10
Associations of Absolute Macronutrient Intake with Cancer Mortality at the 25th, 50th, and 75th Percentile of Intake for Each Macronutrient 11
Macronutrient Composition and Cardiovascular Mortality 12
Macronutrient Composition and Cancer Mortality 13
Macronutrient Composition and Cardiovascular Mortality for Males and Females 14
Macronutrient Composition and Cancer Mortality for Males and Females 15
Macronutrient Composition and All-Cause Mortality: Comorbidity Sensitivity Analysis 16
Macronutrient Composition and All-Cause Mortality: Sensitivity Analysis Including Only Participants with Two Complete 24-hour recalls 17
Macronutrient Composition and Healthy Eating Index 18
Macronutrient Composition and Dietary Fatty Acid Profile 19
Macronutrient Composition and intake of Dietary Fiber, Sugar, and Sodium 20

II. SUPPLEMENTARY TABLES

Model[1] Generalized Additive Model Coefficients for Macronutrient Intake and Mortality 21
Model[2] Generalized Additive Model Coefficients for Macronutrient Intake and Mortality 22
Model[3] Generalized Additive Model Coefficients for Macronutrient Intake and Mortality 23
Generalized Additive Model Coefficients for Individual Macronutrient Percentages and Mortality 24
Generalized Additive Model Coefficients for Macronutrient Intake and Mortality with Interaction for Sex 25
Model Comparisons for With and Without Macronutrients by Sex Interaction 26
Generalized Additive Model Coefficients for Comorbidity Sensitivity 27
Generalized Additive Model Coefficients for Dietary Recall Sensitivity 28

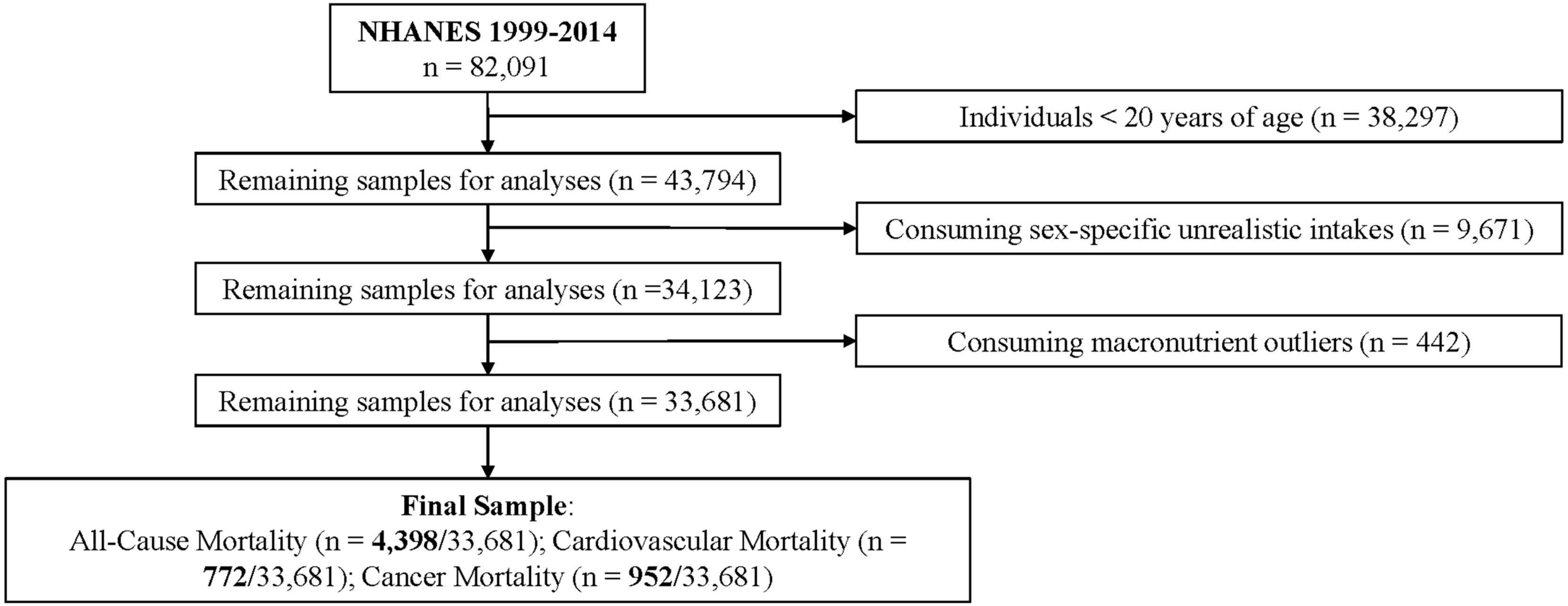

Figure S1. Participant Flowchart.

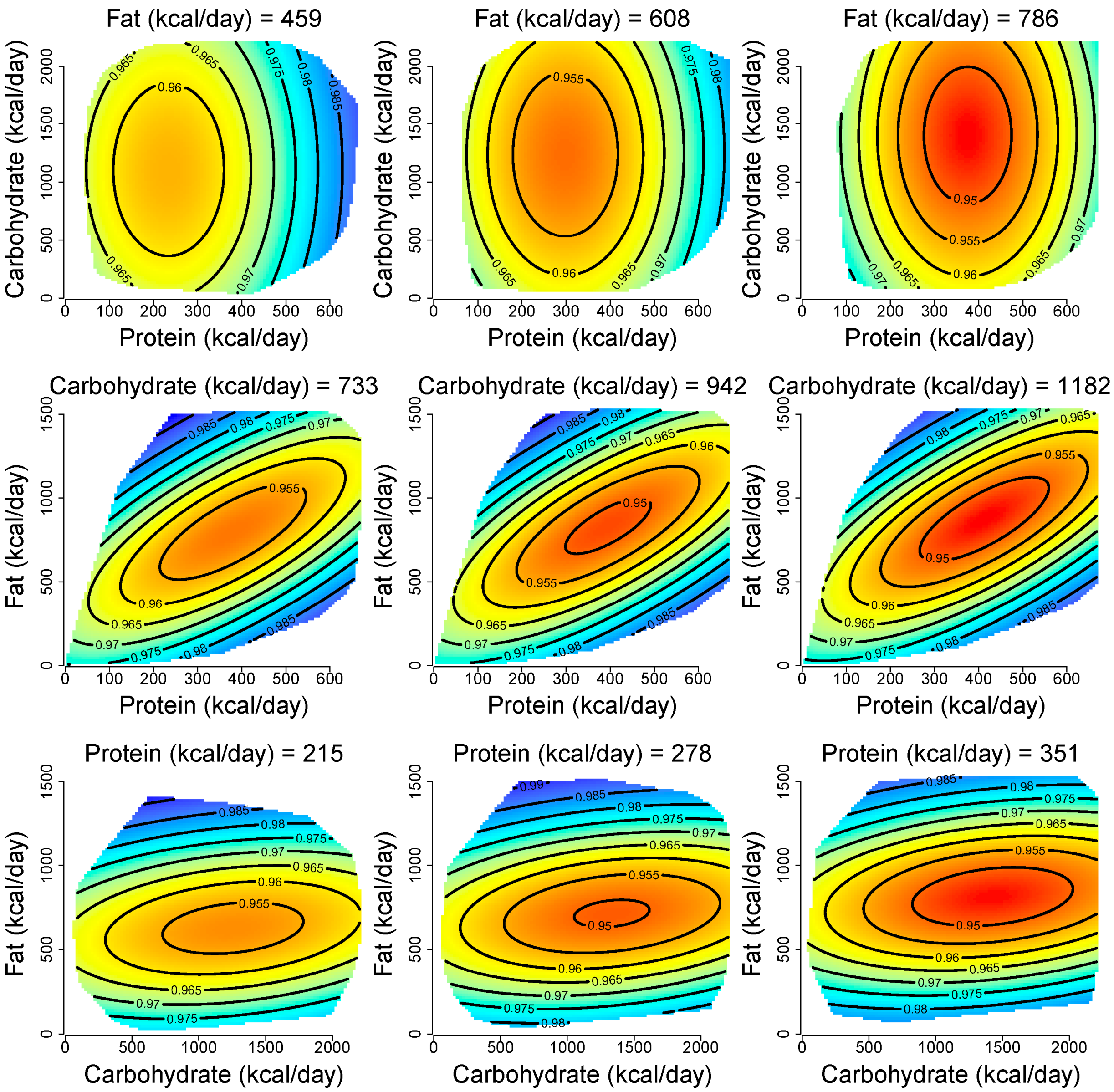

Figure S2. Associations of Absolute Macronutrient Intake with All-Cause Mortality at the 25th, 50th, and 75th Percentile of Intake for Each Macronutrient. Each surface shows the survival function scale for all-cause mortality in a nutrient space of all three macronutrients. The x and y-axis represent two macronutrient exposures sliced through the 25th, 50th, and 75th percentile of the macronutrient shown at the top of each column from left to right respectively. Response values are colored such that warm colors show a higher risk of mortality and cooler colors show a lower mortality risk. Response surfaces were adjusted for age, sex, household income, BMI, physical activity, and healthy eating index.

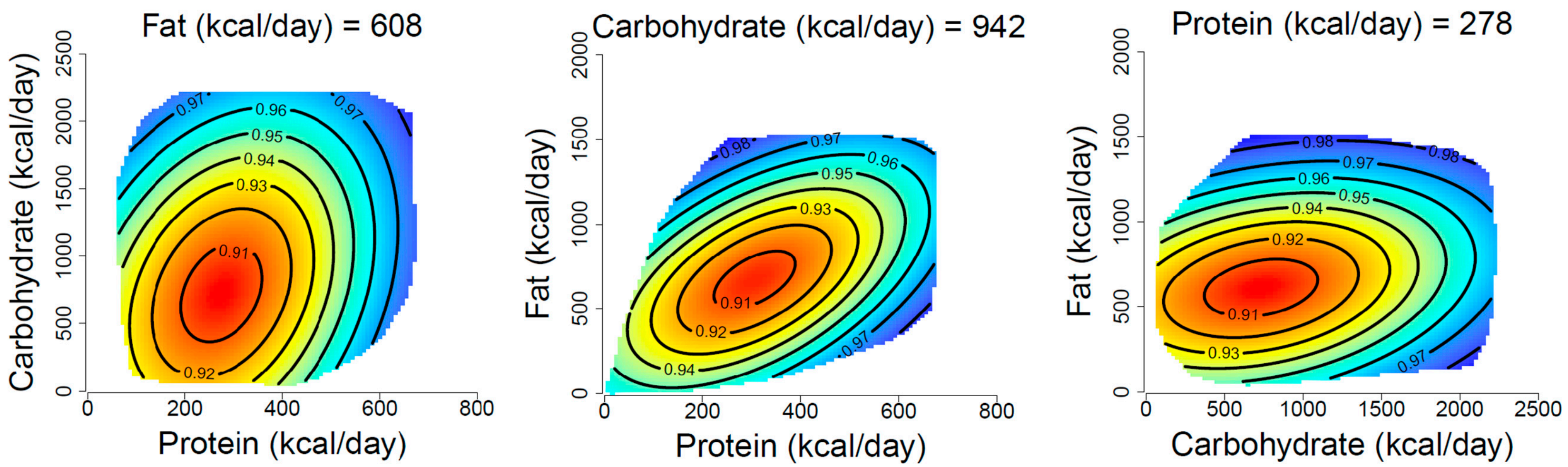

Figure S3. Unadjusted Absolute Macronutrient Intake and All-Cause Mortality. Each surface shows the unadjusted survival function scale for all-cause mortality in a nutrient space of all three macronutrients. The x and y-axis represent two macronutrient exposures sliced through the median value of the macronutrient shown at the top of each figure. Response values are colored such that warm colors show a higher risk of mortality and cooler colors show a lower mortality risk.

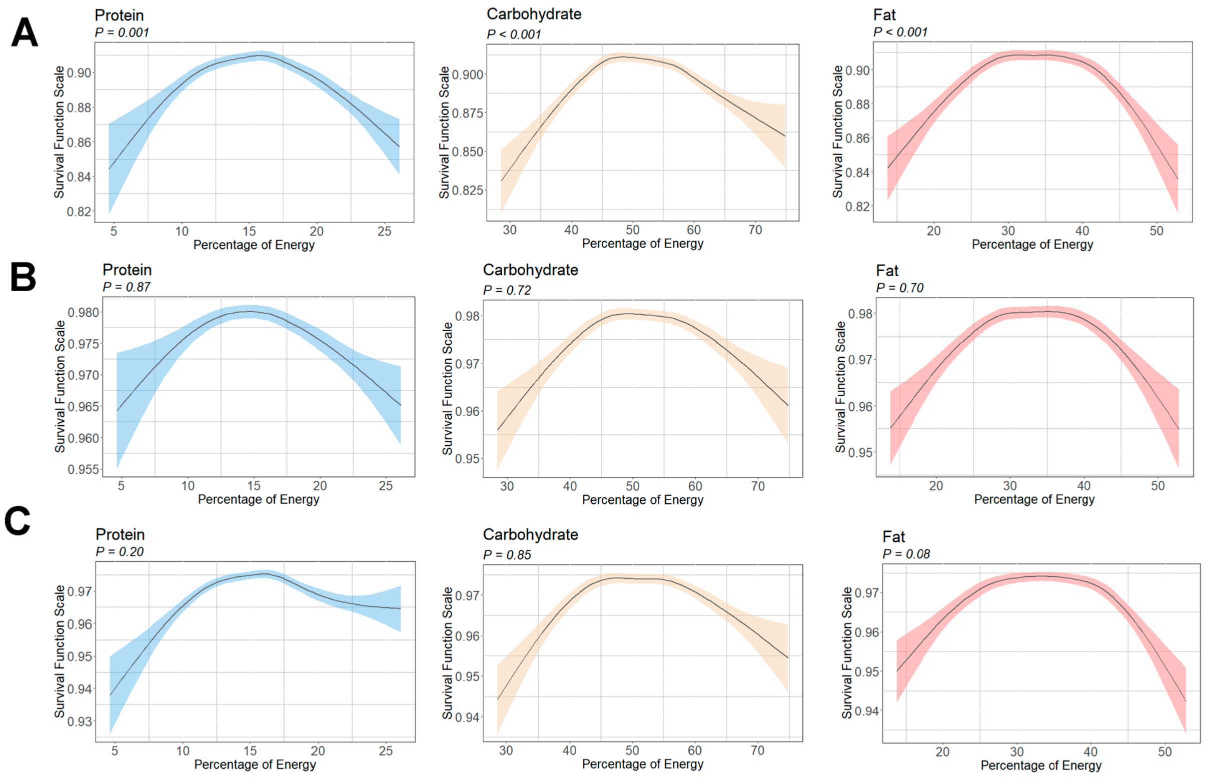

Figure S4. Individual Macronutrient Associations with All-Cause, Cardiovascular, and Cancer Mortality. Plots show the relationship for individual macronutrients as a percentage of total energy intake with the survival function score for all-cause mortality (Row A), cardiovascular mortality (Row B), and cancer mortality (Row C). Relationships were determined by individual generalized additive models adjusted for age, sex, household income, BMI, physical activity, and healthy eating index.

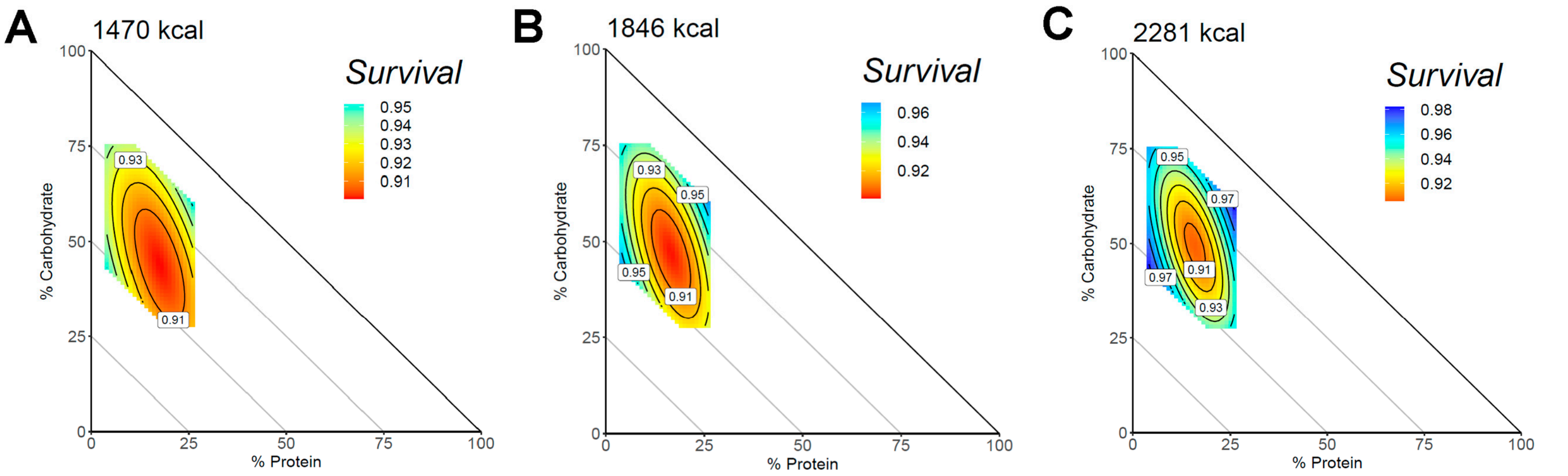

Figure S5. Unadjusted Macronutrient Composition and All-Cause Mortality. The mixture triangle shows the unadjusted model predictions of the all-cause mortality survival score for the range of macronutrient percentages in this dataset. Figure A-C shows the predictions using the 25th, 50th, and 75th percentile of caloric intake for the study population. The x and y-axis show protein and carbohydrate respectively. Percentage of fat can be inferred as decreasing moving away from the origin, such that each point on the triangle can be summed to equal 100%. Response values are colored such that warm colors show a higher risk of mortality and cooler colors show a lower mortality risk.

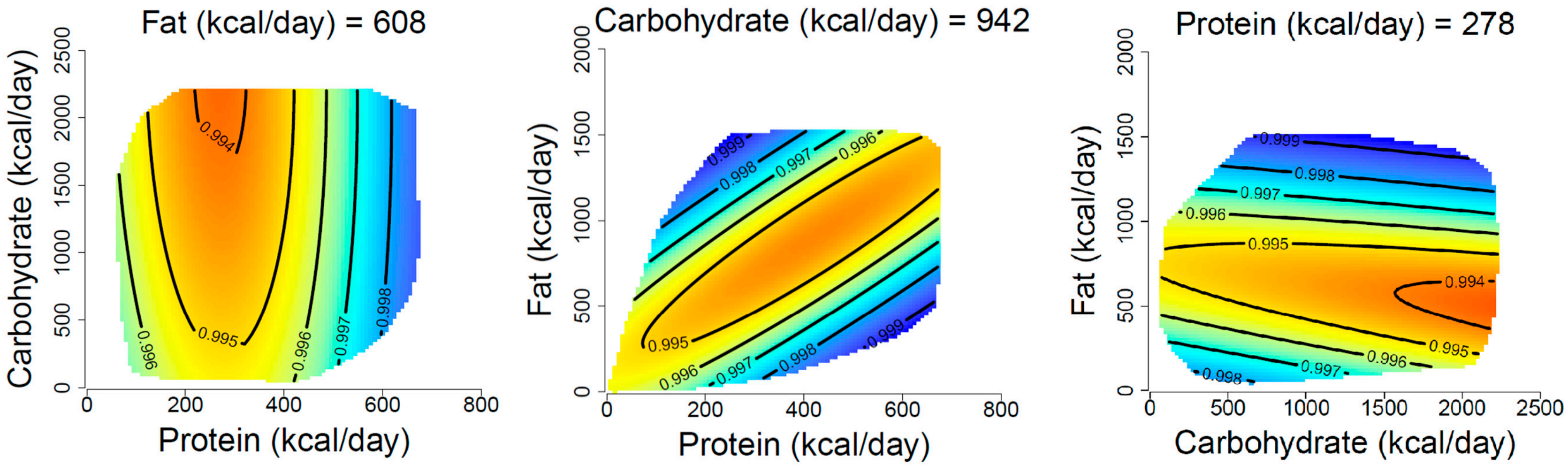

Figure S6. Absolute Macronutrient Intake and Cardiovascular Mortality. Each surface shows the survival function scale for cardiovascular in a nutrient space of all three macronutrients. The x and y-axis represent two macronutrient exposures sliced through the median value of the macronutrient shown at the top of each figure. Response values are colored such that warm colors show a lower risk of mortality and cooler colors show a higher cardiovascular mortality risk. Response surfaces were adjusted for age, sex, household income, BMI, physical activity, and healthy eating index.

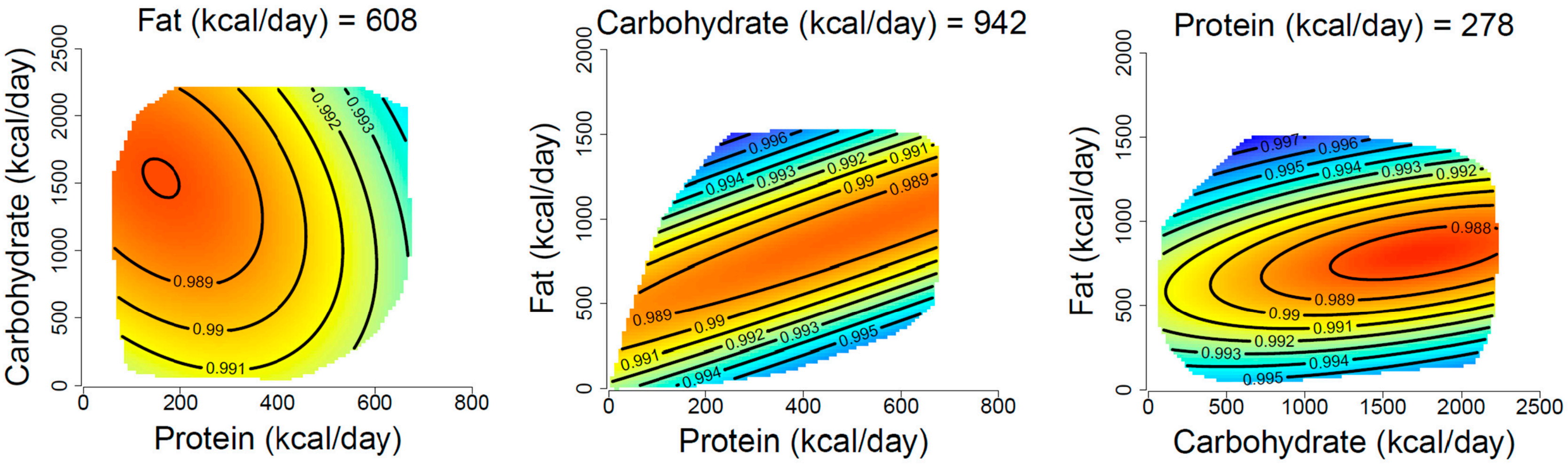

Figure S7. Absolute Macronutrient Intake and Cancer Mortality. Each surface shows the survival function scale for cancer mortality in a nutrient space of all three macronutrients. The x and y-axis represent two macronutrient exposures sliced through the median value of the macronutrient shown at the top of each figure. Response values are colored such that warm colors show a higher risk of mortality and cooler colors show a lower mortality risk. Response surfaces were adjusted for age, sex, household income, BMI, physical activity, and healthy eating index.

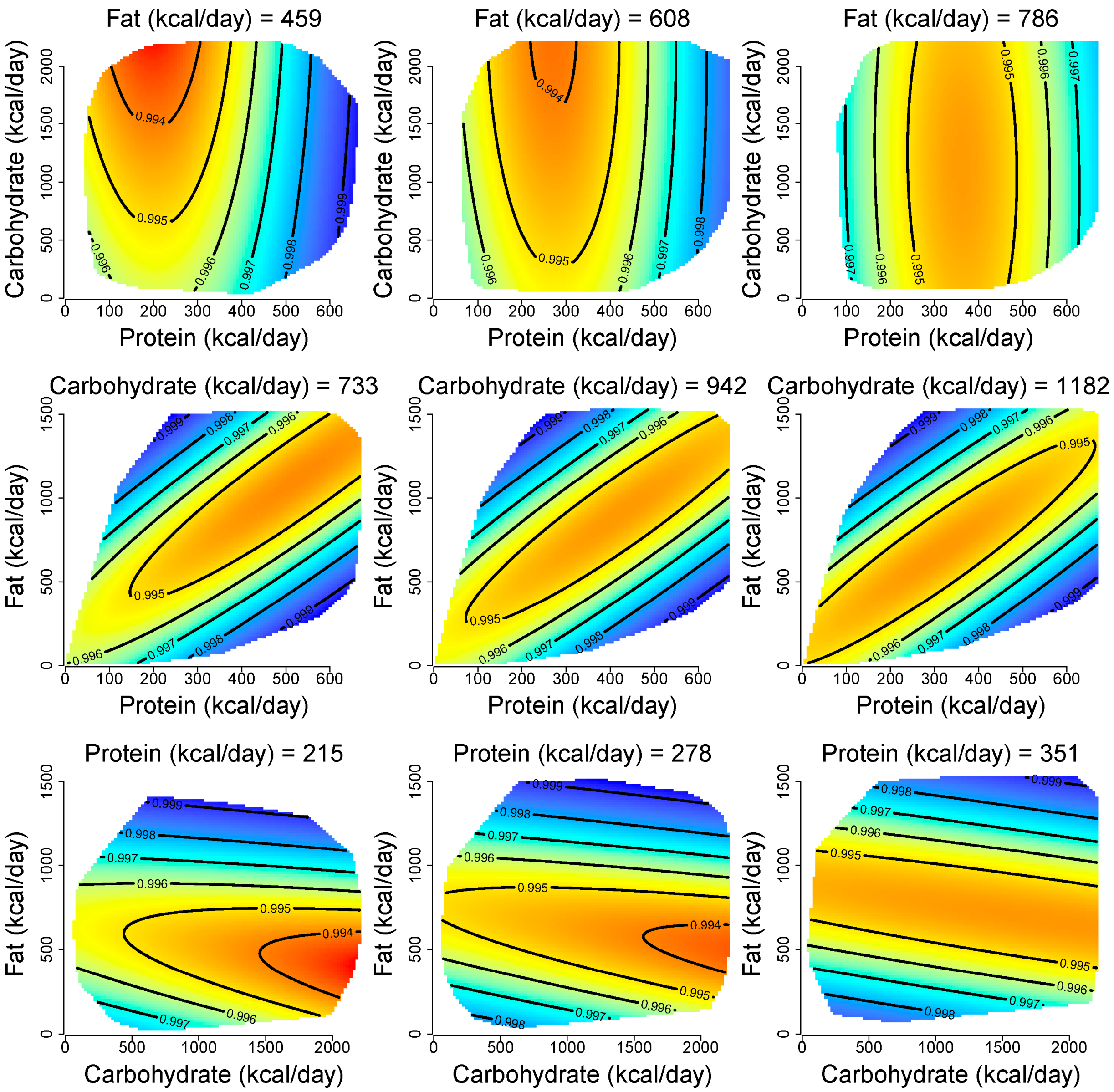

Figure S8. Associations of Absolute Macronutrient Intake with Cardiovascular Mortality at the 25th, 50th, and 75th Percentile of Intake for Each Macronutrient. Each surface shows the survival function scale for cardiovascular mortality in a nutrient space of all three macronutrients. The x and y-axis represent two macronutrient exposures sliced through the 25th, 50th, and 75th percentile of the macronutrient shown at the top of each column from left to right respectively. Response values are colored such that warm colors show a higher risk of mortality and cooler colors show a lower mortality risk. Response surfaces were adjusted for age, sex, household income, BMI, physical activity, and healthy eating index.

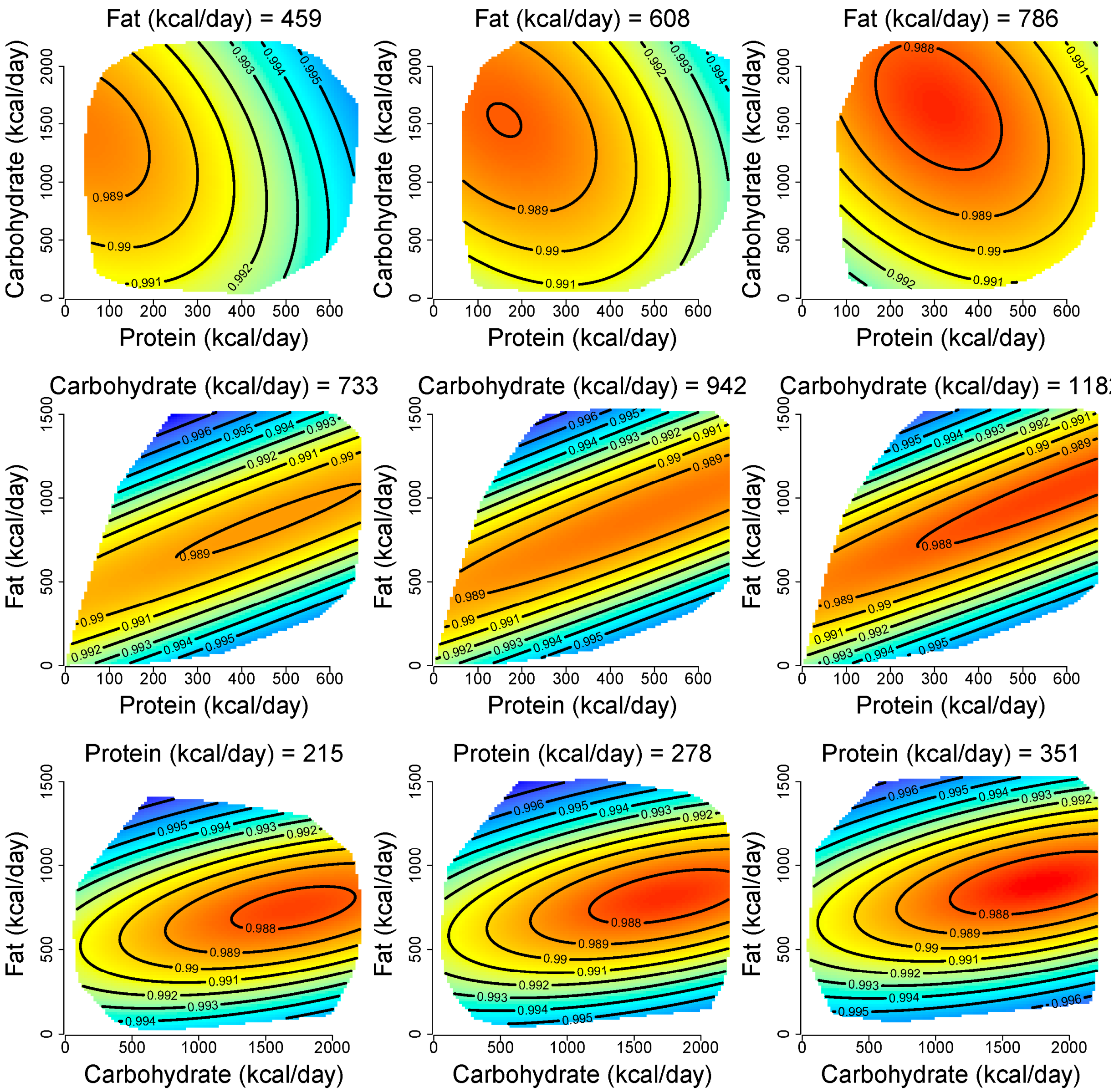

Figure S9. Associations of Absolute Macronutrient Intake with Cancer Mortality at the 25th, 50th, and 75th Percentile of Intake for Each Macronutrient. Each surface shows the survival function scale for cancer mortality in a nutrient space of all three macronutrients. The x and y-axis represent two macronutrient exposures sliced through the 25th, 50th, and 75th percentile of the macronutrient shown at the top of each column from left to right respectively. Response values are colored such that warm colors show a higher risk of mortality and cooler colors show a lower mortality risk. Response surfaces were adjusted for age, sex, household income, BMI, physical activity, and healthy eating index.

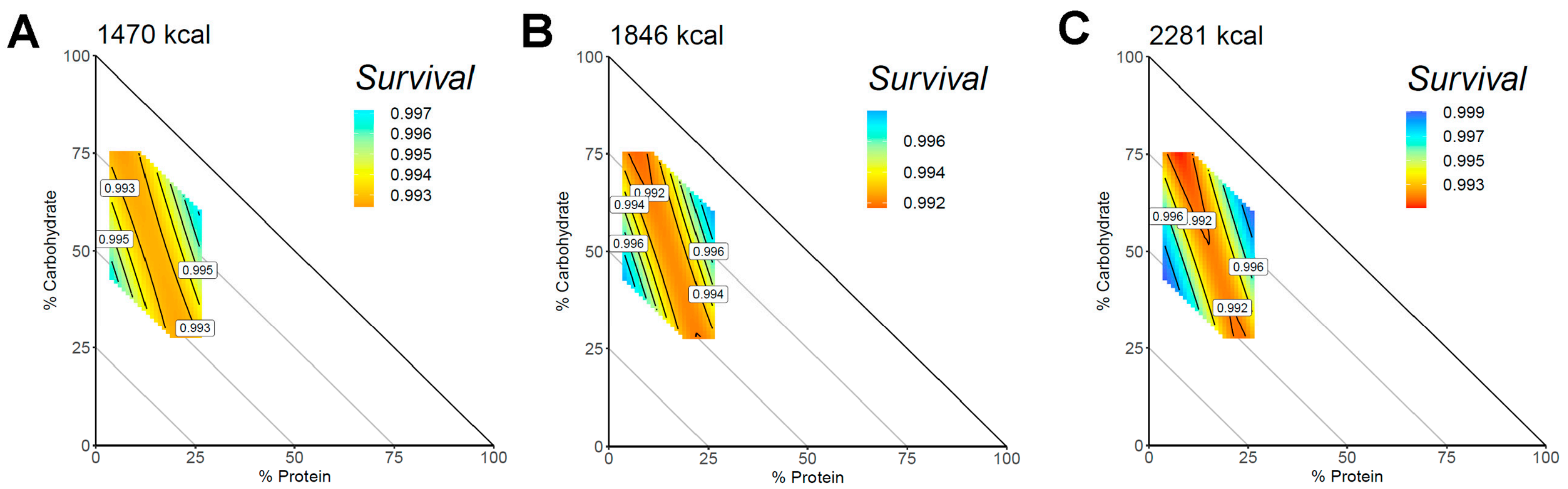

Figure S10. Macronutrient Composition and Cardiovascular Mortality. The mixture triangle shows the model predictions of the survival score for the range of macronutrient percentages in this dataset. Figure A-C shows the predictions using the 25th, 50th, and 75th percentile of caloric intake for the study population. The x and y-axis show protein and carbohydrate respectively. Percentage of fat can be inferred as decreasing moving away from the origin, such that each point on the triangle can be summed to equal 100%. Response values are colored such that warm colors show a higher risk of mortality and cooler colors show a lower mortality risk. Response surfaces were adjusted for age, sex, household income, BMI, physical activity, and healthy eating index.

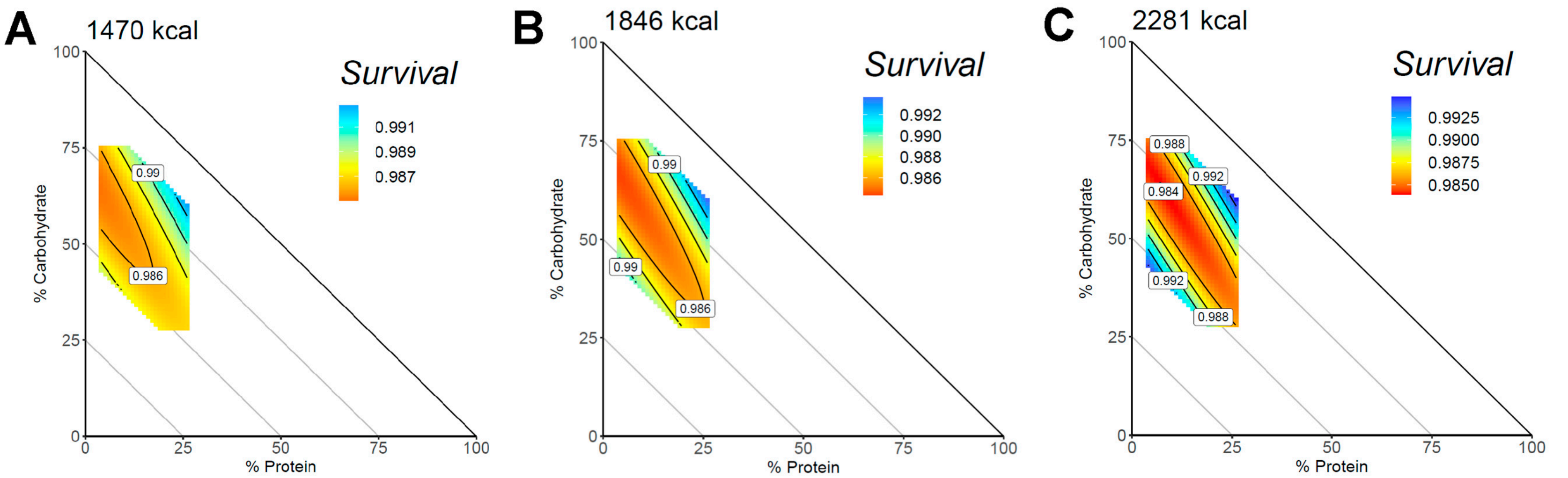

Figure S11. Macronutrient Composition and Cancer Mortality. The mixture triangle shows the model predictions of the cancer mortality survival score for the range of macronutrient percentages in this dataset. Figure A-C shows the predictions using the 25th, 50th, and 75th percentile of caloric intake for the study population. The x and y-axis show protein and carbohydrate respectively. Percentage of fat can be inferred as decreasing moving away from the origin, such that each point on the triangle can be summed to equal 100%. Response values are colored such that warm colors show a higher risk of mortality and cooler colors show a lower mortality risk. Response surfaces were adjusted for age, sex, household income, BMI, physical activity, and healthy eating index.

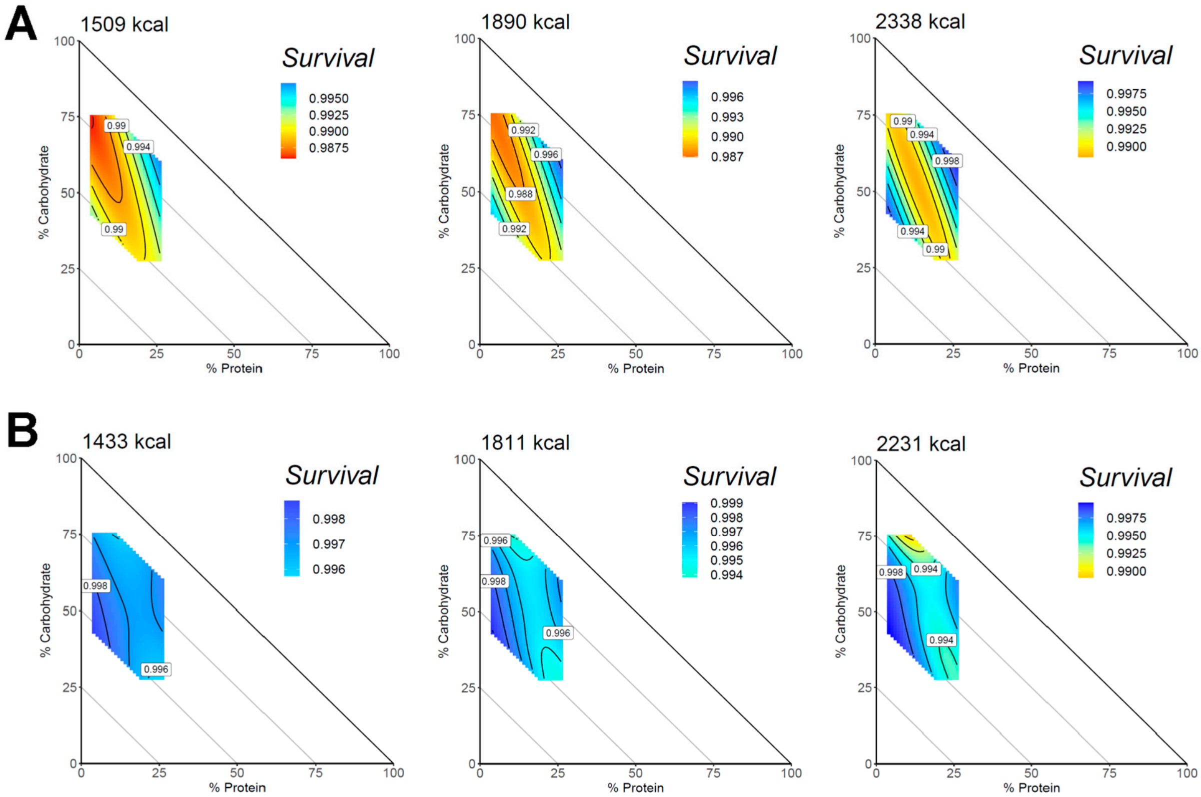

Figure S12. Macronutrient Composition and Cardiovascular Mortality for Males and Females. The mixture triangle shows the model predictions of the cardiovascular mortality survival score for the range of macronutrient percentages in this dataset. Figure A shows the predictions using the 25th, 50th, and 75th percentile of caloric intake for males and Figure B shows this for females. The x and y-axis show protein and carbohydrate respectively. Percentage of fat can be inferred as decreasing moving away from the origin, such that each point on the triangle can be summed to equal 100%. Response values are colored such that warm colors show a higher risk of mortality and cooler colors show a lower mortality risk. Response surfaces were adjusted for age, sex, household income, BMI, physical activity, and healthy eating index.

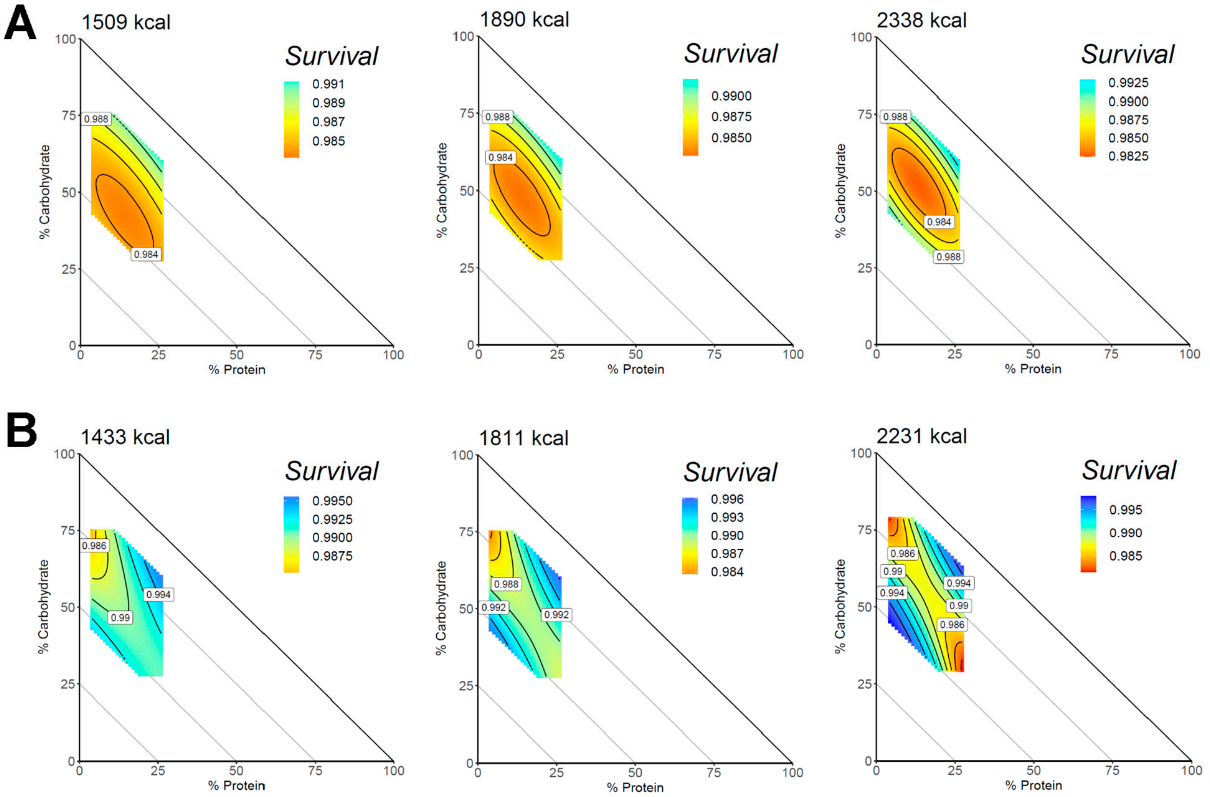

Figure S13. Macronutrient Composition and Cancer Mortality for Males and Females. The mixture triangle shows the model predictions of the cancer mortality survival score for the range of macronutrient percentages in this dataset. Figure A shows the predictions using the 25th, 50th, and 75th percentile of caloric intake for males and Figure B shows this for females. The x and y-axis show protein and carbohydrate respectively. Percentage of fat can be inferred as decreasing moving away from the origin, such that each point on the triangle can be summed to equal 100%. Response values are colored such that warm colors show a higher risk of mortality and cooler colors show a lower mortality risk. Response surfaces were adjusted for age, sex, household income, BMI, physical activity, and healthy eating index.

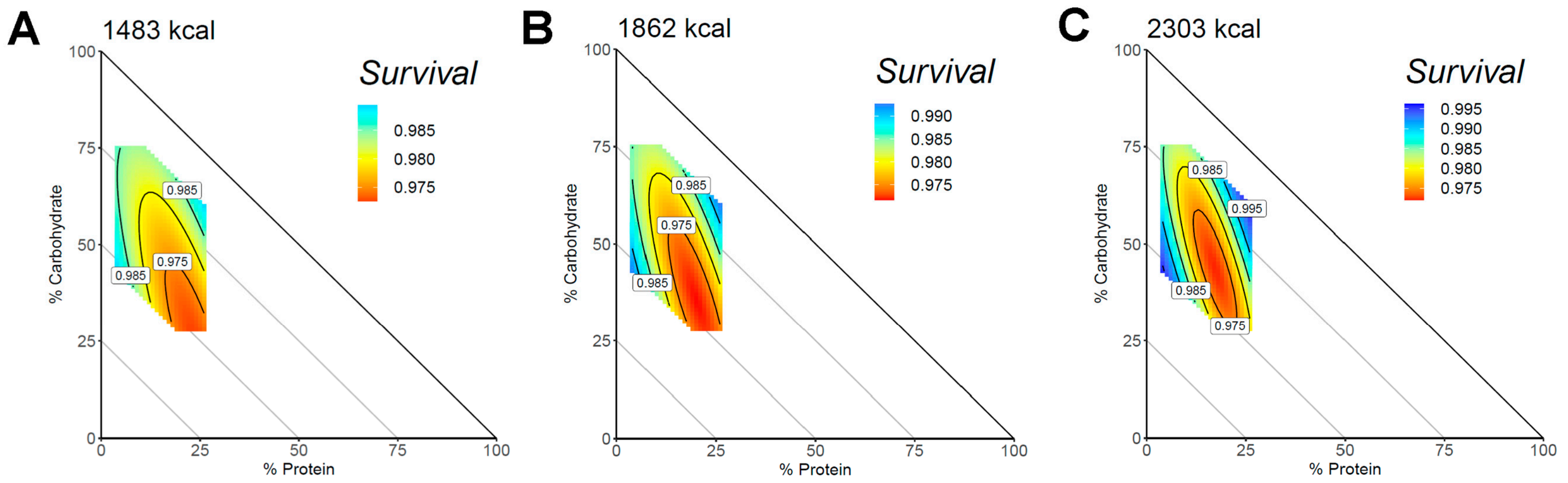

Figure S14. Macronutrient Composition and All-Cause Mortality: Comorbidity Sensitivity Analysis. The mixture triangle shows the model predictions of the all-cause mortality survival score for all-cause mortality within the range of macronutrient percentages in this dataset. Figure A-C shows the predictions using the 25th, 50th, and 75th percentile of caloric intake for the study population. The x and y-axis show protein and carbohydrate respectively. Percentage of fat can be inferred as decreasing moving away from the origin, such that each point on the triangle can be summed to equal 100%. Response values are colored such that warm colors show a higher risk of mortality and cooler colors show a lower mortality risk. Response surfaces were adjusted for age, sex, household income, BMI, physical activity, and healthy eating index.

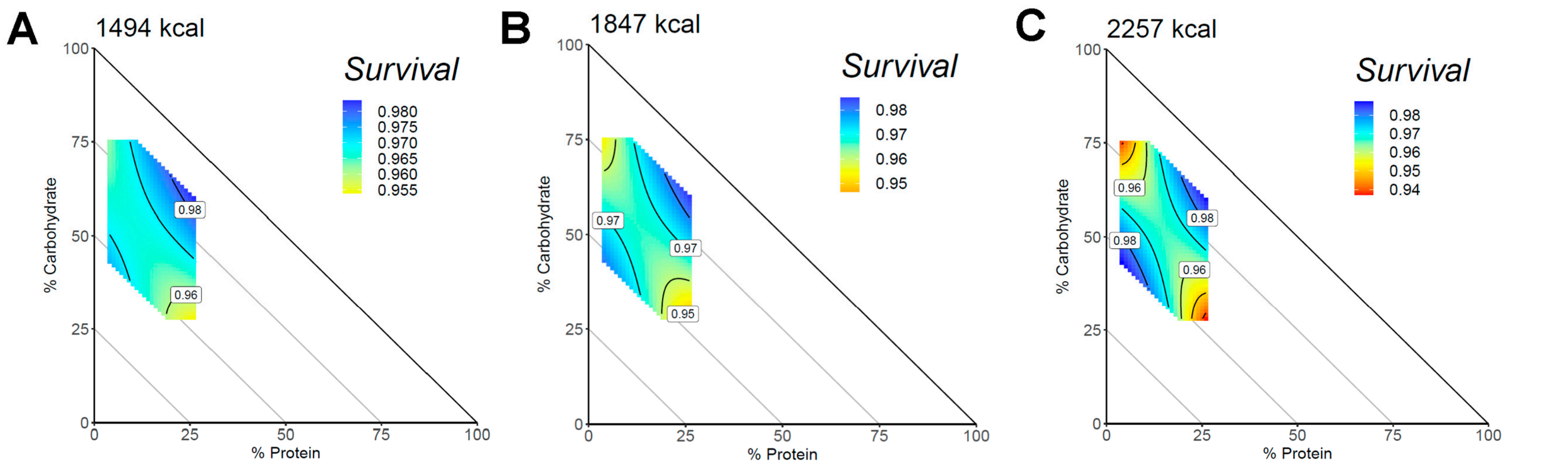

Figure S15. Macronutrient Composition and All-Cause Mortality: Sensitivity Analysis Including Only Participants with Two Complete 24-hour recalls. The mixture triangle shows the model predictions of the all-cause mortality survival score for all-cause mortality within the range of macronutrient percentages in this dataset. Figure A-C shows the predictions using the 25th, 50th, and 75th percentile of caloric intake for the study population. The x and y-axis show protein and carbohydrate respectively. Percentage of fat can be inferred as decreasing moving away from the origin, such that each point on the triangle can be summed to equal 100%. Response values are colored such that warm colors show a higher risk of mortality and cooler colors show a lower mortality risk. Response surfaces were adjusted for age, sex, household income, BMI, physical activity, and healthy eating index.

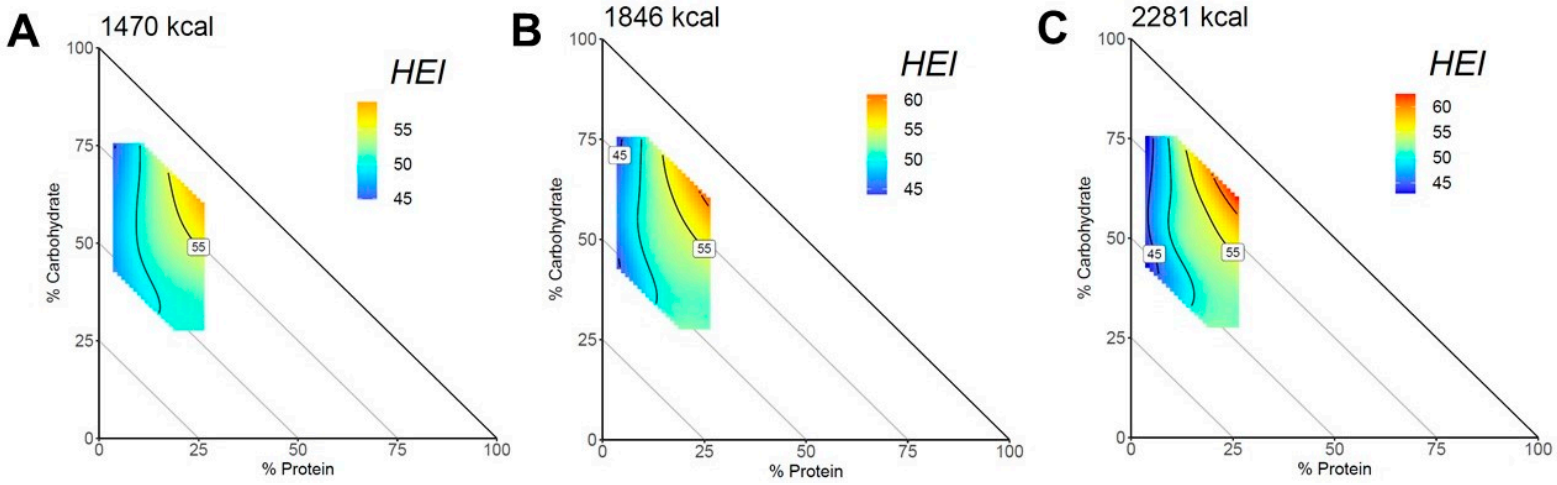

Figure S16. Macronutrient Composition and Healthy Eating Index. The mixture triangle shows the model predictions of the healthy eating index scores for the range of macronutrient percentages in this dataset. Figure A-C shows the predictions using the 25th, 50th, and 75th percentile of caloric intake for the study population. The x and y-axis show protein and carbohydrate respectively. Percentage of fat can be inferred as decreasing moving away from the origin, such that each point on the triangle can be summed to equal 100%. Response surfaces were adjusted for age, sex, household income, BMI, and physical activity.

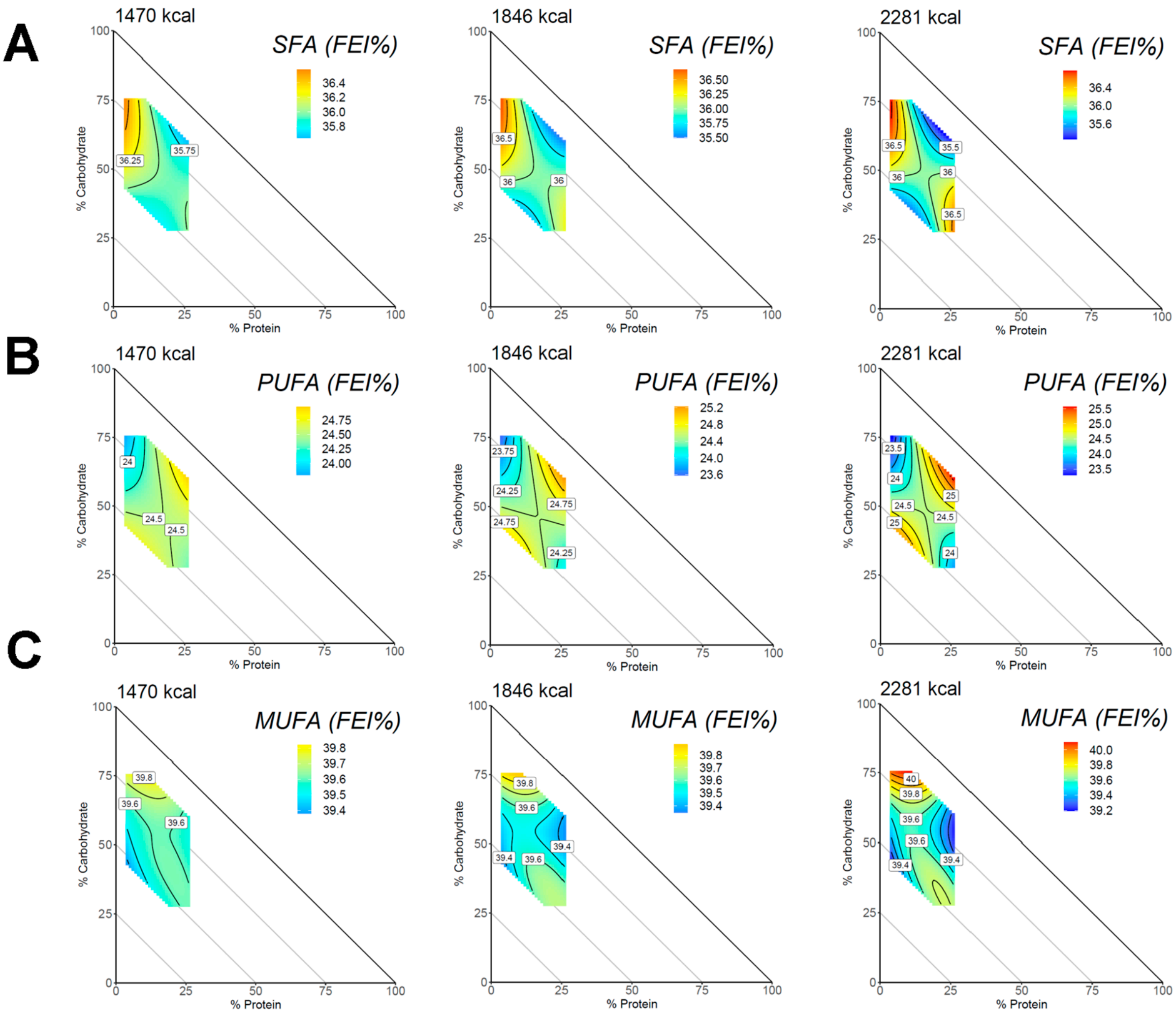

Figure S17. Macronutrient Composition and Dietary Fatty Acid Profile. The mixture triangle shows the model predictions of dietary fatty acid profile for the range of macronutrient percentages in this dataset. Row A shows the predictions of dietary saturated fat (SFA) expressed as a percentage of total fat. The predictions use the 25th, 50th, and 75th percentile of caloric intake for the study cohort from left to right. Row B shows predictions for polyunsaturated fat (PUFA), and Row C shows predictions for monounsaturated fat (MUFA). The x and y-axis show protein and carbohydrate respectively. Percentage of fat can be inferred as decreasing moving away from the origin, such that each point on the triangle can be summed to equal 100%. Response values are colored such that warm colors show a lower risk of mortality and cooler colors show a higher mortality risk. Response surfaces were adjusted for age, sex, household income, BMI, and physical activity. Percentage of total fat intake (FEI%)

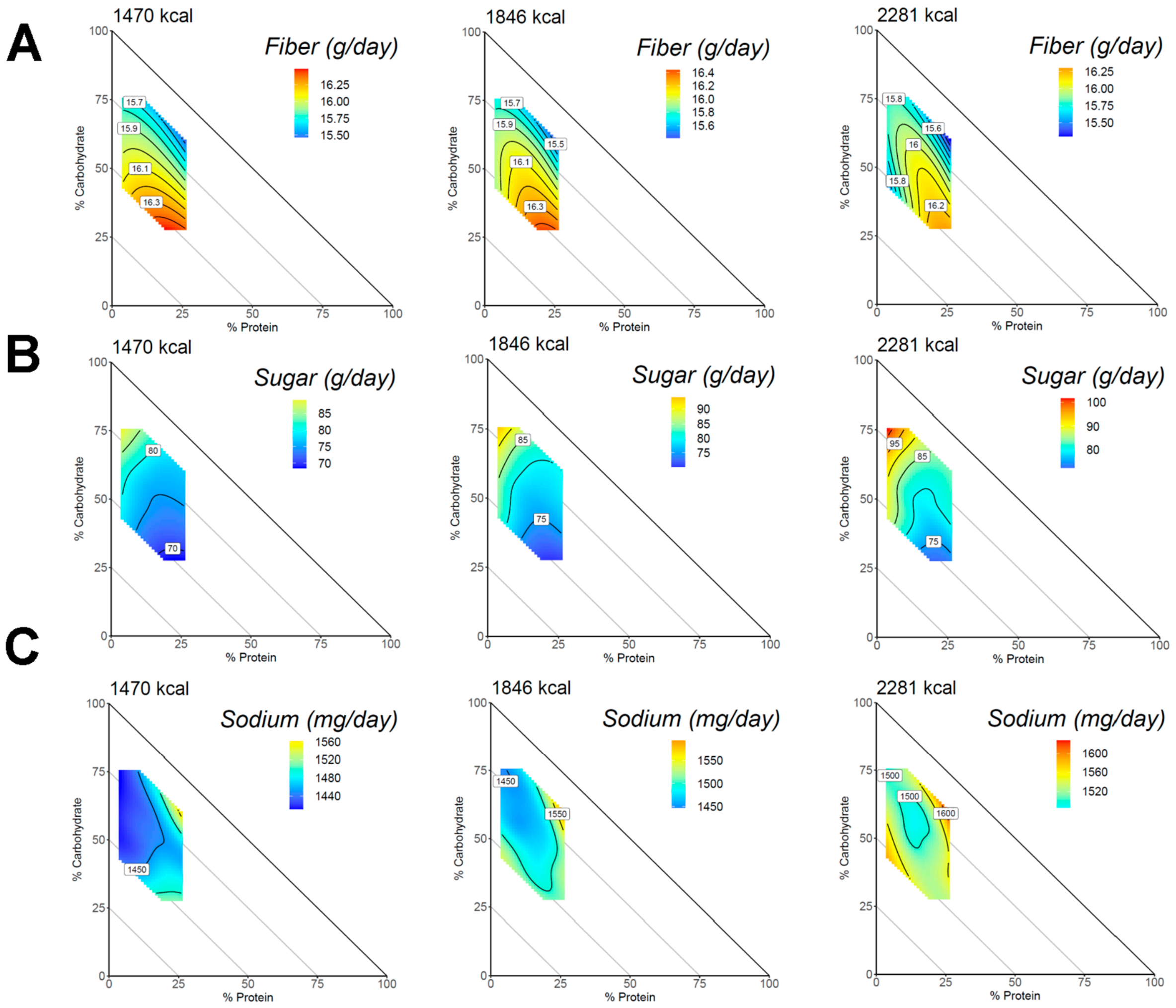

Figure S18. Macronutrient Composition and intake of Dietary Fiber, Sugar, and Sodium. The mixture triangle shows the model predictions of dietary fiber (g/day), sugar (g/day), and sodium (mg/day) for the range of macronutrient percentages in this dataset. Row A shows the predictions of dietary fiber using the 25th, 50th, and 75th percentile of caloric intake for the study cohort from left to right. Row B shows predictions for dietary fiber, and row C shows predictions for sodium. The x and y-axis show protein and carbohydrate respectively. Percentage of fat can be inferred as decreasing moving away from the origin, such that each point on the triangle can be summed to equal 100%. Response values are colored such that warm colors show a lower risk of mortality and cooler colors show a higher mortality risk. Response surfaces were adjusted for age, sex, household income, BMI, and physical activity.

SUPPLEMENTARY TABLES

Table S1. Model[1] Generalized Additive Model Coefficients for Macronutrient Intake and Mortality

Model	edf	Ref.df	Chi.sq	*P* value	Dev Exp	Scale
**All-Cause Mortality**	–	–	–	–	26.9%	1.0
s(Protein, Carbohydrate, Fat)	10.59	11.86	101.4	<0.001	–	–
Age	7.60	8.42	3,877.8	<0.001	–	–
Household Income	1.29	1.52	204.8	<0.001	–	–
Sex	Est: -0.31	SE: 0.03	Z: -10.0	<0.001	–	–
**Cardiovascular Mortality**	–	–	–	–	31.8%	1.0
s(Protein, Carbohydrate, Fat)	9.01	9.01	18.52	0.03	–	–
Age	4.09	5.02	1,221.89	<0.001	–	–
Household Income	1.00	1.00	73.39	<0.001	–	–
Sex	Est: -0.72	SE: 0.08	Z: -9.50	<0.001	–	–
**Cancer Mortality**	–	–	–	–	21.0%	1.0
s(Protein, Carbohydrate, Fat)	9.00	9.01	17.37	0.04	–	–
Age	4.68	5.71	1,046.46	<0.001	–	–
Household Income	4.61	5.63	41.70	<0.001	–	–
Sex	Est: -0.53	SE: 0.07	Z: -7.82	<0.001	–	–

Model outputs for associations of macronutrient intake with all-cause, cardiovascular, and cancer mortality after adjustment for age, sex, and household income are shown above. A significant macronutrient model can be interpreted such that the relationship of each macronutrient with the specified outcome is dependent upon the relative intake of all three macronutrients. Given the complex nonlinearity in these models, specific macronutrient relationships and effect size are best interpreted visually. Effective degrees of freedom (edf); reference degrees of freedom (Ref.df); F statistic (FS); deviance explained (Dev Exp); estimate (Est); standard error (SE); Z score (Z).

Table S2. Model[2] Generalized Additive Coefficients for Macronutrient Intake and Mortality

Model	edf	Ref.df	Chi.sq	*P* value	Dev Exp	Scale
**All-Cause Mortality**	–	–	–	–	27.1%	1.0
s(Protein, Carbohydrate, Fat)	11.26	12.96	106.7	<0.001	–	–
Age	7.62	8.44	3,752.0	<0.001	–	–
Household Income	1.13	1.24	143.6	<0.001	–	–
Sex	Est: -0.31	SE: 0.03	Z: -9.85	<0.001	–	–
Race/Ethnicity	Est: 0.08	SE: 0.01	Z: 5.76	<0.001	–	–
Education	Est: -0.09	SE: 0.02	Z: -4.49	<0.001	–	–
**Cardiovascular Mortality**	–	–	–	–	32.0%	1.0
s(Protein, Carbohydrate, Fat)	9.01	9.02	19.90	0.02	–	–
Age	4.10	5.03	1,184.16	<0.001	–	–
Household Income	1.00	1.00	54.68	<0.001	–	–
Sex	Est: -0.72	SE: 0.08	Z: -9.51	<0.001	–	–
Race/Ethnicity	Est: 0.12	SE: 0.03	Z: 3.58	<0.001	–	–
Education	Est: -0.10	SE: 0.05	Z: -2.19	0.03	–	–
**Cancer Mortality**	–	–	–	–	21.3%	1.0
s(Protein, Carbohydrate, Fat)	9.01	9.02	18.49	0.03	–	–
Age	4.65	5.68	1,000.55	<0.001	–	–
Household Income	4.47	5.47	27.16	<0.001	–	–
Sex	Est: -0.53	SE: 0.07	Z: -7.82	<0.001	–	–
Race/Ethnicity	Est: 0.14	SE: 0.03	Z: 4.61	<0.001	–	–
Education	Est: -0.11	SE: 0.04	Z: -2.64	0.008	–	–

Generalized additive model outputs for associations of macronutrient intake with all-cause, cardiovascular, and cancer mortality after adjustment for age, sex, household income, race/ethnicity, and education are shown above. A significant macronutrient model can be interpreted such that the relationship of each macronutrient with the specified outcome is dependent upon the relative intake of all three macronutrients. Given the complex nonlinearity in these models, specific macronutrient relationships and effect size are best interpreted visually. Effective degrees of freedom (edf); reference degrees of freedom (Ref.df); F statistic (FS); deviance explained (Dev Exp); estimate (Est); standard error (SE); Z score (Z).

Table S3. Model[3] Generalized Additive Model Coefficients for Macronutrient Intake and Mortality

Model	edf	Ref.df	Chi.sq	*P* value	Dev Exp	Scale
**All-Cause Mortality**	–	–	–	–	28.1%	1.0
s(Protein, Carbohydrate, Fat)	11.27	12.97	100.75	<0.001	–	–
s(Age)	7.68	8.48	3,388.26	<0.001	–	–
s(Household Income)	1.18	1.34	102.37	<0.001	–	–
Sex	Est: -0.37	SE: 0.03	Z: -11.31	<0.001	–	–
Race/Ethnicity	Est: 0.07	SE: 0.01	Z: 4.79	<0.001	–	–
Education	Est: -0.05	SE: 0.02	Z: -2.46	0.002	–	–
Alcohol	Est: 0.18	SE: 0.02	Z: 7.73	<0.001	–	–
Smoker	Est: -0.08	SE: 0.03	Z: -3.11	0.002	–	–
s(Physical Activity)	6.34	7.24	117.57	<0.001	–	–
s(Healthy Eating Index)	5.58	6.78	26.14	0.03	–	–
s(Body Mass Index)	5.97	7.14	49.18	<0.001	–	–
**Cardiovascular Mortality**	–	–	–	–	33.1%	1.0
s(Protein, Carbohydrate, Fat)	9.01	9.02	19.16	0.02	–	–
s(Age)	3.72	4.60	1,049.75	<0.001	–	–
s(Household Income)	1.00	1.00	40.97	<0.001	–	–
Sex	Est: -0.77	SE: 0.08	Z: -9.55	<0.001	–	–
Race/Ethnicity	Est: 0.11	SE: 0.03	Z: 3.10	0.002	–	–
Education	Est: -0.06	SE: 0.05	Z: -1.28	0.20	–	–
Alcohol	Est: 0.16	SE: 0.06	Z: 2.87	0.004	–	–
Smoker	Est: -0.13	SE: 0.06	Z: -2.06	0.04	–	–
s(Physical Activity)	5.23	6.10	34.33	<0.001	–	–
s(Healthy Eating Index)	1.55	1.94	1.07	0.62	–	–
s(Body Mass Index)	5.09	14.38	14.38	0.04	–	–
**Cancer Mortality**	–	–	–	–	22.3%	1.0
s(Protein, Carbohydrate, Fat)	9.02	9.04	17.14	0.05	–	–
s(Age)	4.40	5.45	933.87	<0.001	–	–
s(Household Income)	4.53	5.54	19.46	0.002	–	–
Sex	Est: -0.52	SE: 0.07	Z: -7.21	<0.001	–	–
Race/Ethnicity	Est: 0.12	SE: 0.03	Z: 3.89	<0.001	–	–
Education	Est: -0.07	SE: 0.04	Z: -1.61	0.11	–	–
Alcohol	Est: 0.13	SE: 0.05	Z: 2.41	0.02	–	–
Smoker	Est: -0.17	SE: 0.05	Z: -3.07	0.002	–	–
s(Physical Activity)	3.42	4.15	29.73	<0.001	–	–
s(Healthy Eating Index)	1.97	2.51	11.98	0.006	–	–
s(Body Mass Index)	4.09	5.12	11.59	0.04	–	–

Model outputs for associations of macronutrient intake with all-cause, cardiovascular, and cancer mortality after adjustment for age, sex, household income, race/ethnicity, education, and lifestyle factors are shown above. A significant macronutrient model can be interpreted such that the relationship of each macronutrient with the specified outcome is dependent upon the relative intake of all three macronutrients. Given the complex nonlinearity in these models, specific macronutrient relationships and effect size are best interpreted visually. Effective degrees of freedom (edf); reference degrees of freedom (Ref.df); F statistic (FS); deviance explained (Dev Exp); estimate (Est); standard error (SE); Z score (Z).

Table S4. Generalized Additive Model Coefficients for Individual Macronutrient Percentages and Mortality

Model	edf	Ref.df	Chi.sq	*P* value	Dev Exp	Scale
**All-Cause Mortality**						
s(Protein)	3.66	4.59	19.56	0.001	27.9%	1.0
s(Carbohydrate)	3.29	4.14	21.59	<0.001	27.8%	1.0
s(Fat)	3.94	4.92	51.34	<0.001	27.8%	1.0
**Cardiovascular Mortality**						
s(Protein)	1.54	1.93	0.27	0.87	32.8%	1.0
s(Carbohydrate)	1.00	1.01	0.13	0.72	32.8%	1.0
s(Fat)	1.01	1.01	0.16	0.70	32.8%	1.0
**Cancer Mortality**						
s(Protein)	1.00	1.00	1.66	0.20	21.8%	1.0
s(Carbohydrate)	1.01	1.02	0.04	0.85	21.8%	1.0
s(Fat)	2.56	3.25	7.18	0.08	21.8%	1.0

Model outputs for associations of individual macronutrients as a percentage of total energy with all-cause, cardiovascular, and cancer mortality. A significant macronutrient model can be interpreted such that the relationship of each macronutrient with the specified outcome is dependent upon the relative intake of all three macronutrients. Given the complex nonlinearity in these models, specific macronutrient relationships and effect size are best interpreted visually. Macronutrients were evaluated individually as a single smooth term in separate models adjusted for age, sex, household income, BMI, physical activity, and healthy eating index. Effective degrees of freedom (edf); reference degrees of freedom (Ref.df); F statistic (FS); deviance explained (Dev Exp).

Table S5. Generalized Additive Model Coefficients for Macronutrient Intake and Mortality with Interaction for Sex

Model	edf	Ref.df	Chi.sq	*P* value	Dev Exp	Scale
**All-Cause Mortality**	–	–	–	–	27.8%	1.0
s(Protein, Carbohydrate, Fat) Male	10.44	11.60	46.53	<0.001	–	–
s(Protein, Carbohydrate, Fat) Female	9.05	9.09	68.63	<0.001	–	–
Sex	Est: -0.35	SE: 0.03	Z: -10.74	<0.001	–	–
**Cardiovascular Mortality**	–	–	–	–	33.4%	1.0
s(Protein, Carbohydrate, Fat) Male	9.01	9.01	17.00	0.049	–	–
s(Protein, Carbohydrate, Fat) Female	13.43	16.37	22.82	0.13	–	–
Sex	Est: -0.76	SE: 0.08	Z: -9.30	<0.001	–	–
**Cancer Mortality**	–	–	–	–	22.1%	1.0
s(Protein, Carbohydrate, Fat) Male	9.00	9.00	13.38	0.32	–	–
s(Protein, Carbohydrate, Fat) Female	9.00	9.00	13.92	0.13	–	–
Sex	Est: -0.51	SE: 0.07	Z: -7.17	<0.001	–	–

Generalized additive model outputs for associations of macronutrient intake with all-cause, cardiovascular, and cancer mortality after adjustment for age, sex, household income, race/ethnicity, and education are shown above. Each model is presented with sex using the "by" argument. A significant macronutrient model can be interpreted such that the relationship of each macronutrient with the specified outcome is dependent upon the relative intake of all three macronutrients. Effective degrees of freedom (edf); reference degrees of freedom (Ref.df); F statistic (FS); deviance explained (Dev Exp); estimate (Est); standard error (SE); Z score (Z).

Table S6. Model Comparisons for With and Without Macronutrients by Sex Interaction

Model	AIC	Dev Exp
All-Cause Mortality	78,385.9	28.1%
All-Cause Mortality With Sex Interaction	78,385.0	28.1%
Cardiovascular Mortality	12,554.9	33.3%
Cardiovascular Mortality With Sex Interaction	12,561.6	33.4%
Cancer Mortality	16,381.6	22.3%
Cancer Mortality With Sex Interaction	16,391.8	22.1%

Model outputs for associations of macronutrient intake with all-cause, cardiovascular, and cancer mortality with and without an interaction term for sex in the fully adjusted model. Akaike Information Criterion scores (AIC) are shown as an indicator of model fitness where lower values have a better overall fit. A difference in AIC of >2 was considered evidence of a difference in model fit. Deviance explained (Dev Exp).

Table S7. Generalized Additive Model Coefficients for Comorbidity Sensitivity

Model	edf	Ref.df	Chi.sq	*P* value	Dev Exp	Scale
**All-Cause Mortality**	–	–	–	–	27.1%	1.0
s(Protein, Carbohydrate, Fat)	9.01	9.03	45.83	<0.001	–	–
s(Age)	6.26	7.38	1,646.76	<0.001	–	–
s(Household Income)	1.00	1.00	38.72	<0.001	–	–
Sex	Est: -0.43	SE: 0.06	Z: -7.42	<0.001	–	–
Race/Ethnicity	Est: 0.05	SE: 0.02	Z: 2.28	0.02	–	–
Education	Est: -0.11	SE: 0.03	Z: -3.29	0.001	–	–
Alcohol	Est: 0.12	SE: 0.04	Z: 2.94	0.003	–	–
Smoker	Est: -0.02	SE: 0.04	Z: -0.53	0.60	–	–
s(Physical Activity)	4.48	5.50	22.24	<0.001	–	–
s(Healthy Eating Index)	5.02	6.17	18.51	0.007	–	–
s(Body Mass Index)	1.21	1.40	11.20	0.004	–	–
**Cardiovascular Mortality**	–	–	–	–	36.1%	1.0
s(Protein, Carbohydrate, Fat)	9.13	9.26	16.98	0.05	–	–
s(Age)	2.17	2.73	423.32	<0.001	–	–
s(Household Income)	1.00	1.00	11.84	<0.001	–	–
Sex	Est: -0.71	SE: 0.16	Z: -4.56	<0.001	–	–
Race/Ethnicity	Est: 0.05	SE: 0.06	Z: 0.82	0.41	–	–
Education	Est: -0.08	SE: 0.09	Z: -0.90	0.37	–	–
Alcohol	Est: 0.11	SE: 0.11	Z: 0.99	0.32	–	–
Smoker	Est: 0.009	SE: 0.11	Z: 0.08	0.94	–	–
s(Physical Activity)	2.71	3.31	10.17	0.02	–	–
s(Healthy Eating Index)	2.76	3.52	3.09	0.37	–	–
s(Body Mass Index)	3.24	4.05	6.96	0.14	–	–
**Cancer Mortality**	–	–	–	–	22.0%	1.0
s(Protein, Carbohydrate, Fat)	9.01	9.01	14.33	0.11	–	–
s(Age)	1.00	1.00	449.42	<0.001	–	–
s(Household Income)	1.36	1.63	3.58	0.08	–	–
Sex	Est: -0.48	SE: 0.12	Z: -4.10	<0.001	–	–
Race/Ethnicity	Est: 0.14	SE: 0.05	Z: 2.95	0.003	–	–
Education	Est: -0.19	SE: 0.07	Z: -2.58	0.01	–	–
Alcohol	Est: -0.02	SE: 0.09	Z: -0.17	0.86	–	–
Smoker	Est: 0.02	SE: 0.08	Z: 0.22	0.83	–	–
s(Physical Activity)	2.58	3.26	6.87	0.09	–	–
s(Healthy Eating Index)	2.02	2.58	2.00	0.49	–	–
s(Body Mass Index)	1.04	1.08	5.09	0.03	–	–

Model outputs for associations of macronutrient intake with all-cause, cardiovascular, and cancer mortality excluding those who reported having a comorbid condition or consuming related medications at the time of the interview. A significant macronutrient model can be interpreted such that the relationship of each macronutrient with the specified outcome is dependent upon the relative intake of all three macronutrients. Given the complex nonlinearity in these models, specific macronutrient relationships and effect size are best interpreted visually. Effective degrees of freedom (edf); reference degrees of freedom (Ref.df); F statistic (FS); deviance explained (Dev Exp); estimate (Est); standard error (SE); Z score (Z).

Table S8. Generalized Additive Model Coefficients for Dietary Recall Sensitivity

Model	edf	Ref.df	Chi.sq	*P* value	Dev Exp	Scale
**All-Cause Mortality**	–	–	–	–	25.1%	1.0
s(Protein, Carbohydrate, Fat)	9.92	10.70	20.70	0.03	–	–
s(Age)	5.22	6.29	1,993.26	<0.001	–	–
s(Household Income)	1.02	1.04	82.73	<0.001	–	–
Sex	Est: -0.48	SE: 0.05	Z: -9.49	<0.001	–	–
Race/Ethnicity	Est: 0.09	SE: 0.02	Z: 3.98	<0.001	–	–
Education	Est: -0.07	SE: 0.03	Z: -2.19	0.03	–	–
Alcohol	Est: 0.12	SE: 0.04	Z: 2.92	0.004	–	–
Smoker	Est: -0.04	SE: 0.04	Z: -1.06	0.29	–	–
s(Physical Activity)	9.92	10.70	20.70	0.03	–	–
s(Healthy Eating Index)	1.22	1.41	19.16	<0.001	–	–
s(Body Mass Index)	4.32	5.39	42.39	<0.001	–	–
**Cardiovascular Mortality**	–	–	–	–	26.8%	1.0
s(Protein, Carbohydrate, Fat)	9.43	9.83	4.30	0.93	–	–
s(Age)	5.07	6.08	482.45	<0.001	–	–
s(Household Income)	1.00	1.00	23.64	<0.001	–	–
Sex	Est: -1.04	SE: 0.13	Z: -7.95	<0.001	–	–
Race/Ethnicity	Est: 0.15	SE: 0.06	Z: 2.55	0.01	–	–
Education	Est: -0.03	SE: 0.07	Z: -0.39	0.70	–	–
Alcohol	Est: 0.06	SE: 0.10	Z: 0.62	0.54	–	–
Smoker	Est: -0.04	SE: 0.09	Z: -0.41	0.68	–	–
s(Physical Activity)	4.28	4.72	18.37	0.001	–	–
s(Healthy Eating Index)	1.00	1.00	3.66	0.06	–	–
s(Body Mass Index)	1.53	1.91	3.44	0.23	–	–
**Cancer Mortality**	–	–	–	–	24.0%	1.0
s(Protein, Carbohydrate, Fat)	9.00	9.01	11.54	0.24	–	–
s(Age)	1.00	1.00	391.57	<0.001	–	–
s(Household Income)	1.00	1.00	10.68	0.001	–	–
Sex	Est: -0.53	SE: 0.11	Z: -4.83	<0.001	–	–
Race/Ethnicity	Est: 0.16	SE: 0.05	Z: 3.13	0.002	–	–
Education	Est: -0.07	SE: 0.07	Z: -1.07	0.29	–	–
Alcohol	Est: 0.08	SE: 0.08	Z: 0.09	0.41	–	–
Smoker	Est: -0.08	SE: 0.08	Z: -0.98	0.33	–	–
s(Physical Activity)	2.57	3.14	12.02	0.009	–	–
s(Healthy Eating Index)	1.00	1.00	6.93	0.008	–	–
s(Body Mass Index)	2.63	3.40	6.17	0.12	–	–

Model outputs for associations of macronutrient intake with all-cause, cardiovascular, and cancer mortality excluding individuals who only reported a single 24-hour dietary recall. Effective degrees of freedom (edf); reference degrees of freedom (Ref.df); F statistic (FS); deviance explained (Dev Exp); estimate (Est); standard error (SE); Z score (Z).
